# Cross-variability decoding for motor imagery EEG signals: a comprehensive review

**DOI:** 10.3389/fnins.2026.1899645

**Published:** 2026-09-08

**Authors:** Lijun Wang, Yueying Zhou, Pengpai Wang, Xiujin Ma, Shufeng Zhou

**Affiliations:** 1 School of Mathematics and Systems Science, Liaocheng University, Liaocheng, China; 2 College of Computer and Information Engineering, Nanjing Tech University, Nanjing, China

**Keywords:** brain-computer interface, cross-variability, electroencephalogram, motor imagery, transfer learning

## Abstract

Motor Imagery-based (MI) Electroencephalography (EEG) has emerged as a leading solution in non-invasive Brain-Computer Interface (BCI) systems, leveraging its strong motor intention correlation to enable reliable neural decoding. However, practical implementation of MI confronts three persistent challenges: low signal-to-noise ratio, substantial variability across subjects or over time, and inherent signal nonstationarity. These fundamental limitations continue to hinder the widespread adoption and operational reliability of MI BCI systems. Despite advances in cross-variability decoding methods, there is a lack of systematic syntheses to guide technological evolution in MI BCI. To address these challenges, this review presents a comprehensive taxonomy of MI EEG cross-variability decoding studies from 2020 to 2025, systematically organizing advances in deep learning and transfer learning. We critically evaluate core algorithmic approaches, including Convolutional Neural Networks (CNN), transformers, feature alignment, domain adaptation, and meta-learning. We then explore the underlying mechanisms of these methods and assess their efficacy across key variability paradigms (mainly cross-subject and cross-session scenarios). Finally, we summarize key findings, highlight unresolved challenges, and outline promising future research directions. These advancements hold significant potential to bridge the gap between laboratory-based MI and real-world clinical and consumer applications.

## 1 Introduction

As a bridge connecting the human brain to electronic devices, Brain-Computer Interface (BCI) innovation establishes uninterrupted interaction through recording and interpretation of neural activity, bypassing traditional neuromuscular interfaces (Wolpaw et al., 2000; Wolpaw et al., 2002). Over the past decade, BCI has found widespread applications spanning medical rehabilitation (Qiu et al., 2023), affective computing (Gong et al., 2023), attention detection (Sun et al., 2025), and workload state detection (Zhou et al., 2022), with continuous technological maturation. Owing to the high temporal resolution, low cost, and ease of acquisition, Electroencephalogram (EEG) signals exhibit significant potential in BCI research (Zhou et al., 2022).

**Figure 1:**
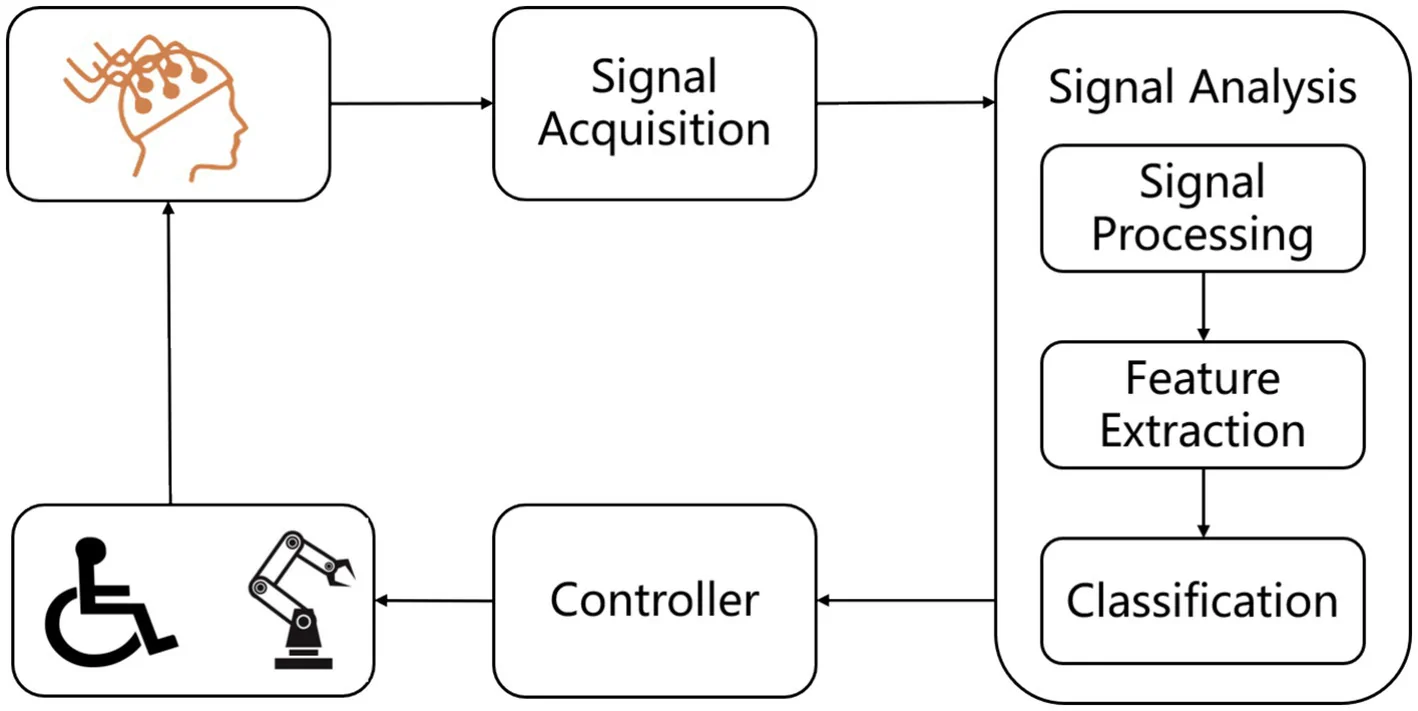
Flowchart of MI BCI system.

The widely used BCI systems include Steady-State Visual Evoked Potential (SSVEP) protocol (Friman et al., 2007), Motor Imagery (MI) decoding (Pfurtscheller and Neuper, 2001), and P300 paradigms (Sutton et al., 1965). In MI BCI systems, cerebral cortical signals are recorded and analyzed to enable external device control through MI decoding (Pfurtscheller and Neuper, 2001). Compared with conventional BCI systems, MI BCI requires neither physical movement nor external stimuli, thereby providing a direct communication channel for patients with motor impairments. A typical MI BCI system, as illustrated in Figure 1, includes three main steps: (1) EEG collection, (2) EEG analysis (which involves preprocessing, feature extraction, and classification), and (3) control of external devices.

**Figure 2:**
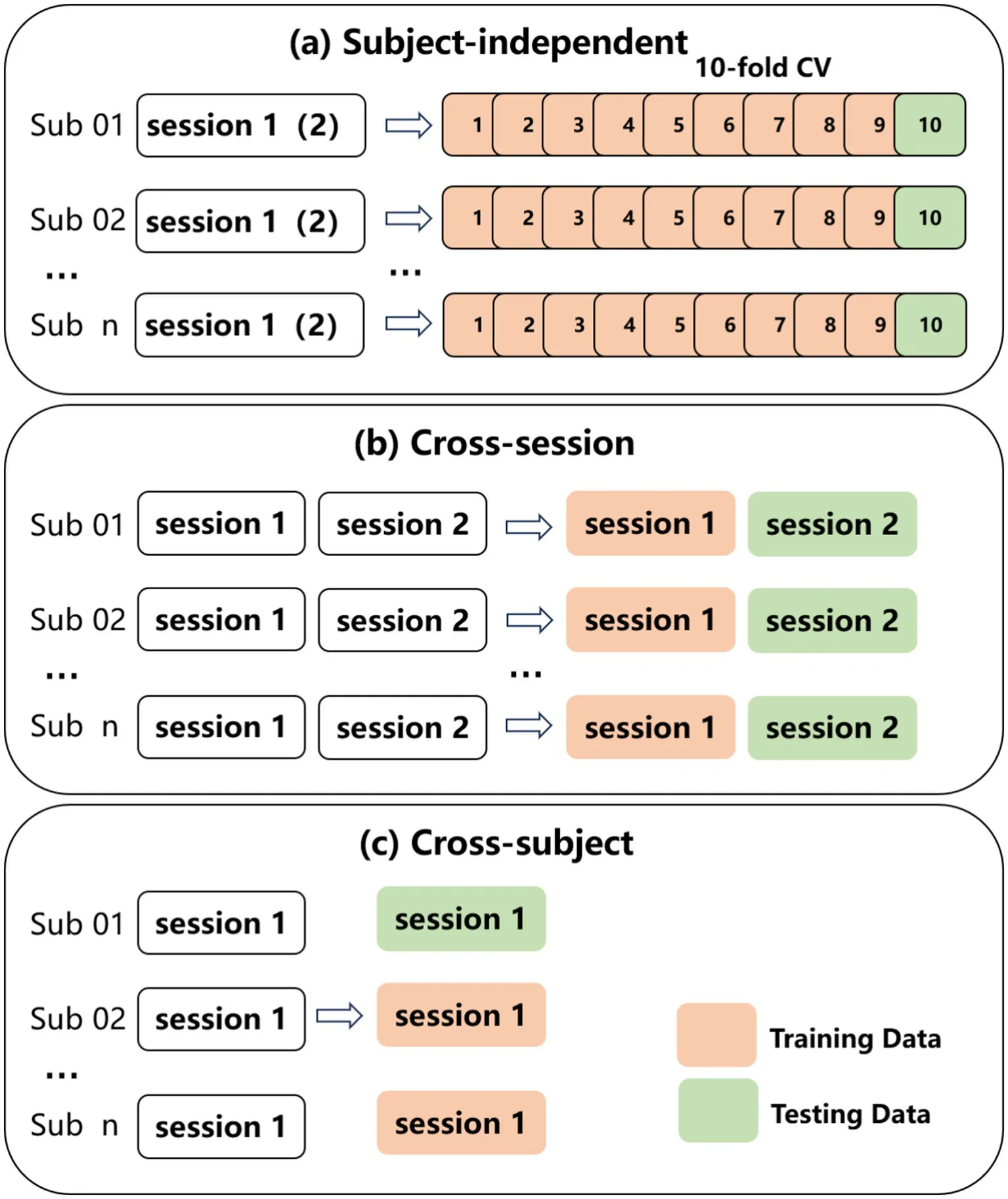
Cross-variability paradigms: **(a)** subject-independent, **(b)** cross-session, **(c)** cross-subject.

BCI has advanced subject-independent MI EEG decoding, which focuses on constructing personalized models for a single subject, as displayed in Figure 2a. Nevertheless, EEG signals present inherent challenges, including high sensitivity to noise and substantial inter-subject diversity. These inconsistencies stem from three primary sources: neurophysiological differences among individuals, variations in acquisition devices, and task-induced dynamic neural pattern variations (Berkhout and Walter, 1968). Crucially, intra-subject variability persists even when the same subject performs identical tasks using consistent equipment across multiple sessions (Gramfort et al., 2013), highlighting the complexity of BCI signal stability. These multifaceted variability sources constitute the fundamental challenge in BCI systems. Therefore, to address subject-independent models’ limitations and enhance generalization, researchers are gradually shifting to cross-variability paradigms. Cross-variability refers to the variations in MI EEG signals across different subjects, sessions, and datasets, which limit model generalization (Wang et al., 2025a; Fan et al., 2025).

The cross-variability paradigm encompasses two principal dimensions: cross-session and cross-subject paradigms (Wu et al., 2022). The cross-session paradigm examines intra-subject variability by analyzing EEG signals collected from the same subject across multiple sessions (Ma et al., 2022). Meanwhile, the cross-subject paradigm utilizes multi-subject EEG datasets to address neurophysiological divergences between participants (Zhou et al., 2025). As delineated in Figure 2, their difference is structured as follows: (a) Subject-dependent: Focus on constructing personalized models for individuals. For instance, EEG data from one session is divided into 10 parts, where each part is alternately used as the training set while the remaining as the test set. (b) Cross-session: Focus on decoding generalization across different periods. Here, the first session of data collected from one subject is used as the training part, and the second session serves as the test part. (c) Cross-subject: Targets inter-subject variability and generalization across multiple subjects. In this paradigm, data from one subject is used as the test set, while data from other subjects serve as the training set.

Before 2020, MI EEG decoding had established a relatively mature technical foundation. Traditional approaches mainly relied on feature representation methods such as Common Spatial Pattern (CSP) (Ramoser et al., 2000), Filter Bank Common Spatial Pattern (FBCSP) (Ang et al., 2008), and Riemannian geometry (Barachant et al., 2012). Among them, Riemannian geometry-based methods enhance the robustness of EEG feature representations by exploiting the manifold structure of covariance matrices. They provide an effective feature representation approach to mitigate distribution shifts caused by differences across subjects and recording sessions. Subsequently, deep learning was gradually introduced into EEG decoding. Shallow/Deep ConvNet (Schirrmeister et al., 2017) demonstrated the effectiveness of end-to-end feature learning with CNNs, while EEGNet (Lawhern et al., 2018) further achieved a lightweight architecture and cross-paradigm generalization. In addition, standardized benchmarking platforms such as MOABB (Jayaram and Barachant, 2018) facilitate fair comparisons among algorithms by providing unified datasets and evaluation protocols. These platforms also highlight the importance of dataset characteristics, cross-variability settings, and evaluation protocols in performance comparisons, providing a foundation for subsequent cross-subject and cross-session generalization.

With the development of MI EEG decoding research, the focus has gradually shifted from improving classification performance in a single scenario to enhancing model generalization across different subjects, sessions, and data distributions. Existing review studies have provided valuable references for understanding the development of MI EEG decoding and the evolution of transfer learning methods in EEG-based BCIs. For example, Al-Qaysi et al. (2021) systematically reviewed intelligent training frameworks for MI EEG and their applications in virtual reality-based BCIs. However, their study mainly focused on system design and application scenarios, with limited discussion on cross-variability decoding. Wu et al. (2022) summarized transfer learning methods in the EEG BCI field from a migration perspective, covering various transfer scenarios, including cross-subject, cross-session, cross-device, and cross-task settings. However, this review mainly focused on general EEG BCI transfer learning and involved multiple EEG paradigms. It lacked a targeted analysis of model design, adaptation mechanisms, and evaluation strategies for cross-variability MI EEG decoding. Li et al. (2026) systematically reviewed the applications of deep learning methods in cross-subject EEG generalization. This review mainly focused on cross-subject generalization problems and covered various EEG tasks. However, it did not provide a targeted analysis of different variation sources, transfer adaptation mechanisms, and practical deployment conditions in cross-variability decoding of MI EEG. Therefore, we systematically summarize deep learning- and transfer learning-based MI EEG cross-variability decoding methods published from 2020 to 2025. Unlike existing reviews, we focus on analyzing key techniques, adaptation mechanisms, and evaluation strategies under different cross-variability scenarios. It further discusses current challenges and future directions to provide a systematic reference for MI EEG cross-variability decoding research.

We structure our work in the following order. Section 2 systematically outlines methodological framework for literature identification, specifying database retrieval strategies. Section 3 provides an in-depth analysis of recent developments in deep learning for MI EEG cross-variability classification through three critical dimensions: cross-subject, cross-session, and hybrid paradigms. Section 4 expands the analysis to transfer learning advancements, classifying existing works into transfer learning, domain adaptation, and other frameworks. Section 5 evaluates persistent technical barriers and emerging research directions, while Section 6 synthesizes key findings and elucidates their significant implications for BCI system optimization.

## 2 Materials and methods

### 2.1 Literature search

**Figure 3:**
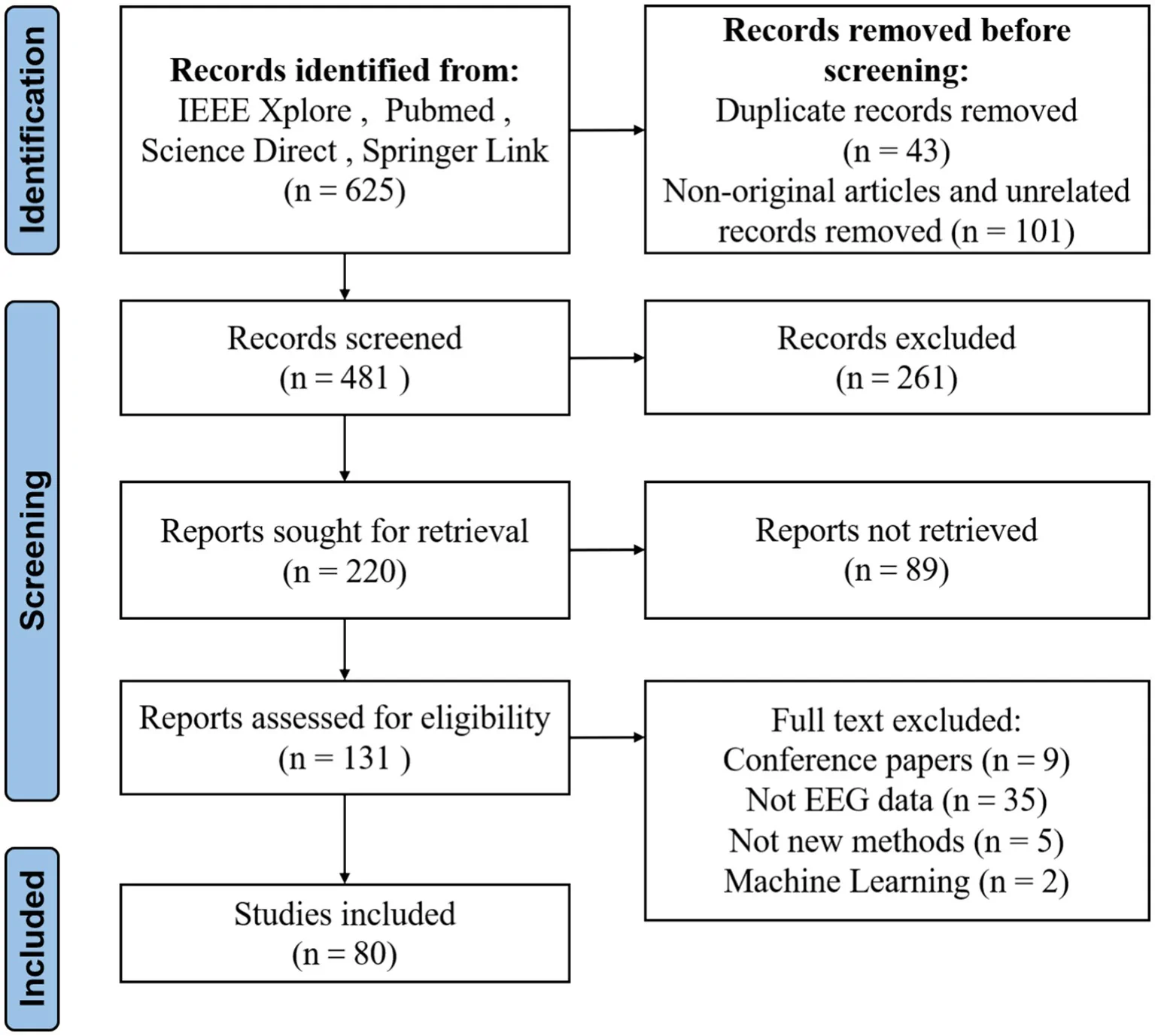
The article search strategy.

Following systematic analysis protocols and PRISMA guidelines, we performed a comprehensive literature search across four major academic databases: IEEE Xplore, PubMed, ScienceDirect, and Springer. The search strategy targeted article titles, abstracts, and keywords. Figure 3 presents a schematic overview of the search strategy.

The database search was performed on October 1, 2025. Searches were conducted in IEEE Xplore, PubMed, ScienceDirect, and Springer Link using predefined combinations of keywords related to cross-variability, transfer learning, deep learning, EEG, and motor imagery. Database-specific search strategies, including the detailed Boolean expressions and searched fields, are provided in Supplementary Table S1. A publication date filter was applied to include studies published from January 1, 2020, to October 1, 2025.

The duplicate records were removed using EndNote X9 before the screening procedure. Following the elimination of duplicate records and studies using only non-public datasets, the remaining publications were rigorously screened through a two-stage screening process. First, two independent reviewers performed title and abstract screening to identify potentially eligible studies. Then, the full texts of the remaining articles were independently evaluated according to the predefined inclusion criteria. Any disagreements between the two reviewers during the screening process were discussed and resolved with the involvement of a third reviewer. The eligibility criteria were as follows: (1) Peer-reviewed articles published in English journals; (2) Studies analyzing MI EEG data; (3) Publications proposing complete and novel modeling frameworks; (4) Publications proposing deep learning or transfer learning based modeling frameworks for MI EEG decoding; (5) Implementation of cross-variability validation and testing methodologies. The final systematic review incorporated a total of 80 eligible studies, of which 21 adopted deep learning approaches, and 59 employed transfer learning techniques.

To improve the transparency and reproducibility of this systematic review, we provided detailed information on the search strategy and data extraction process in the supplementary materials. The excluded studies during the full-text screening process and the corresponding reasons for exclusion are summarized in Supplementary Table S2. The extracted data from the included studies are provided in Supplementary Table S3.

**Figure 4:**
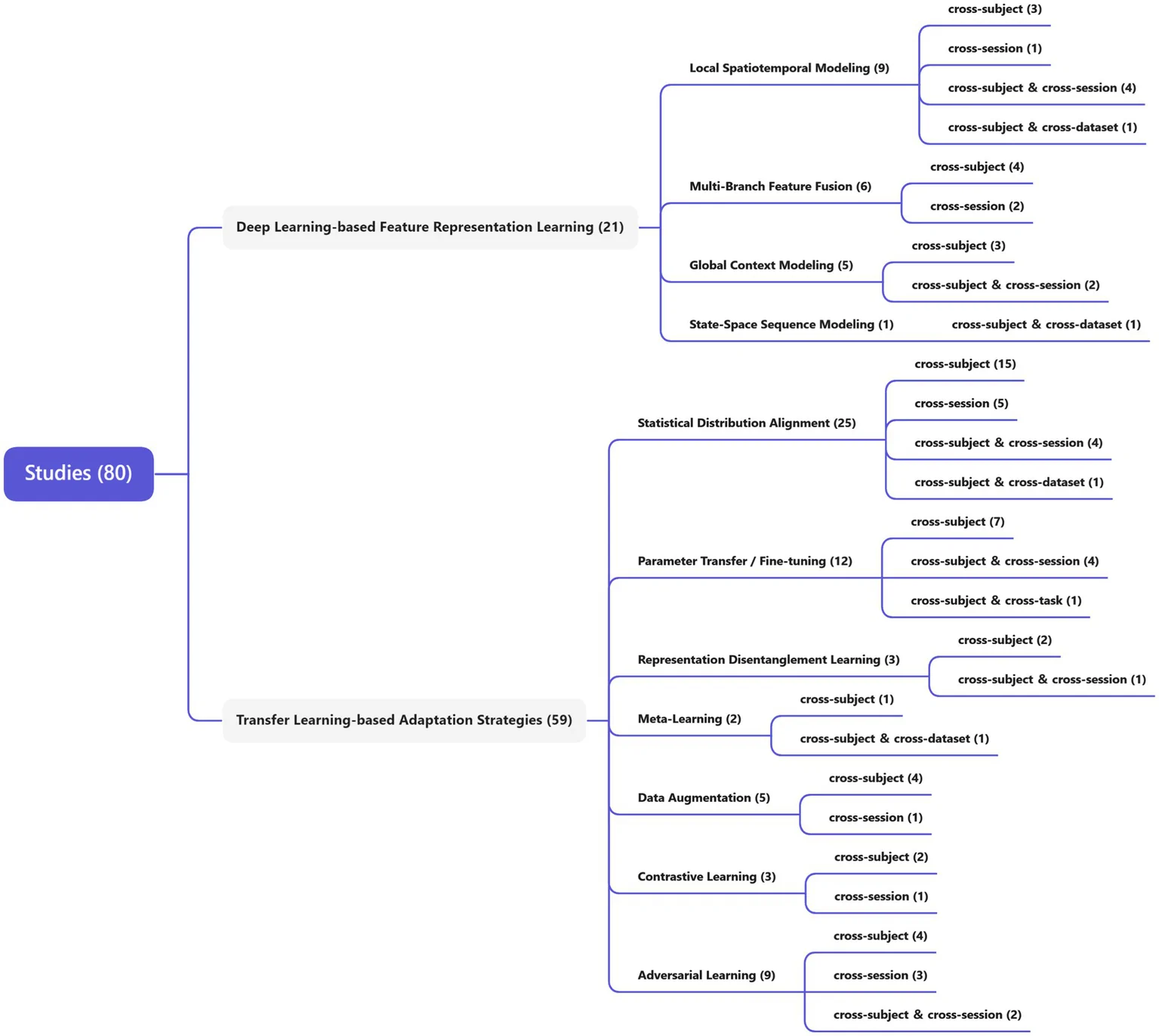
Structured diagram of the classification framework.

We reorganize MI EEG cross-variability decoding methods according to their learning mechanisms, as shown in Figure 4. Existing approaches are mainly divided into two categories: deep learning-based feature representation methods and transfer learning-based adaptation strategies. Deep learning-based methods aim to learn robust EEG representations. According to their representation mechanisms, they are further categorized into local spatiotemporal modeling, multi-branch feature fusion, global context modeling, and state-space sequence modeling. Transfer learning-based methods aim to reduce domain discrepancies through knowledge transfer and adaptation strategies. These strategies include statistical distribution alignment, parameter transfer/fine-tuning, representation disentanglement learning, meta-learning, data augmentation, contrastive learning, and adversarial learning. These methods address different variability sources, including cross-subject, cross-session, cross-dataset, and cross-task variations, by considering different learning objectives, alignment spaces, and target-data conditions. This taxonomy provides a mechanism-oriented view of existing methods and clarifies their roles in addressing MI EEG cross-variability challenges.

### 2.2 Preliminary results

**Figure 5:**
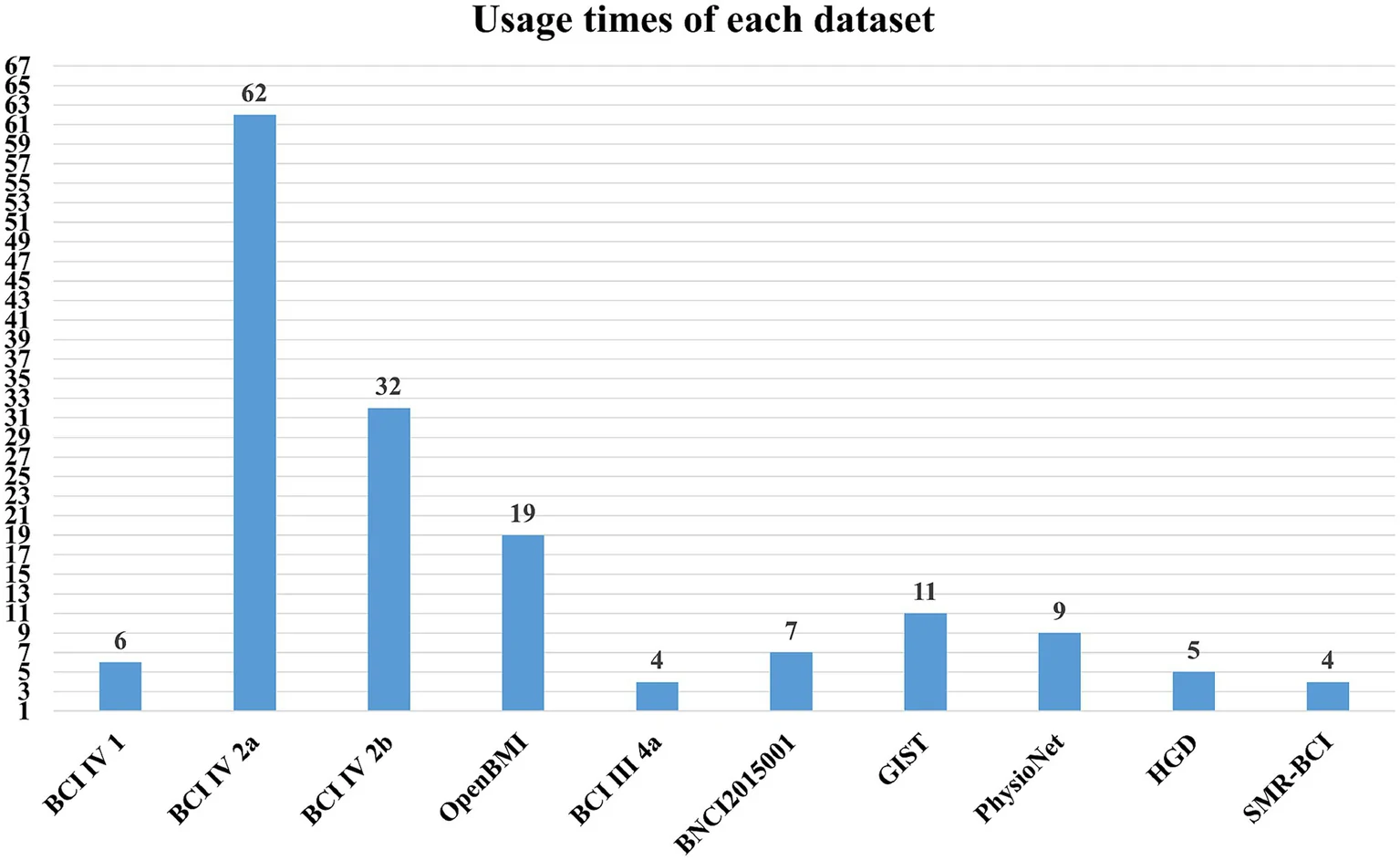
Usage times of each dataset.

We tallied the public datasets from 80 papers, and the results are presented in Figure 5. The top six most frequently used public datasets for MI are BCI IV 2a (Naeem et al., 2006), BCI IV 2b (Leeb et al., 2007), OpenBMI (Lee et al., 2019), GIST (Cho et al., 2017), PhysioNet (Goldberger et al., 2000), and BNCI2015001 (Faller et al., 2012). Table 1 displays key details for each dataset, including number of subjects, sessions, categories, channels acquired, and sampling frequency.

**Table 1:** Introduction to common datasets.

Dataset	Sub	Ses	Class	Channel	Frequency
BCI IV 2a	9	2	LH, RH, F, T	22	250 Hz
BCI IV 2b	9	5	LH, RH	3	250 Hz
OpenBMI	54	2	LH, RH	62	1,000 Hz
GIST Ses 1	52		LH, RH	64	512 Hz
PhysioNet	109	3	LH, RH, BH, F	64	160 Hz
BNCI2015001	12	3	RH, F	13	512 Hz

^1^ ‘Sub’, subject; ‘Ses’, session.

^2^ In class column, ‘LH’, ‘RH’, ‘F’, ‘T’, ‘BH’, denote left-hand, right-hand, feet, tongue, and both-hand classification task, respectively.

**Figure 6:**
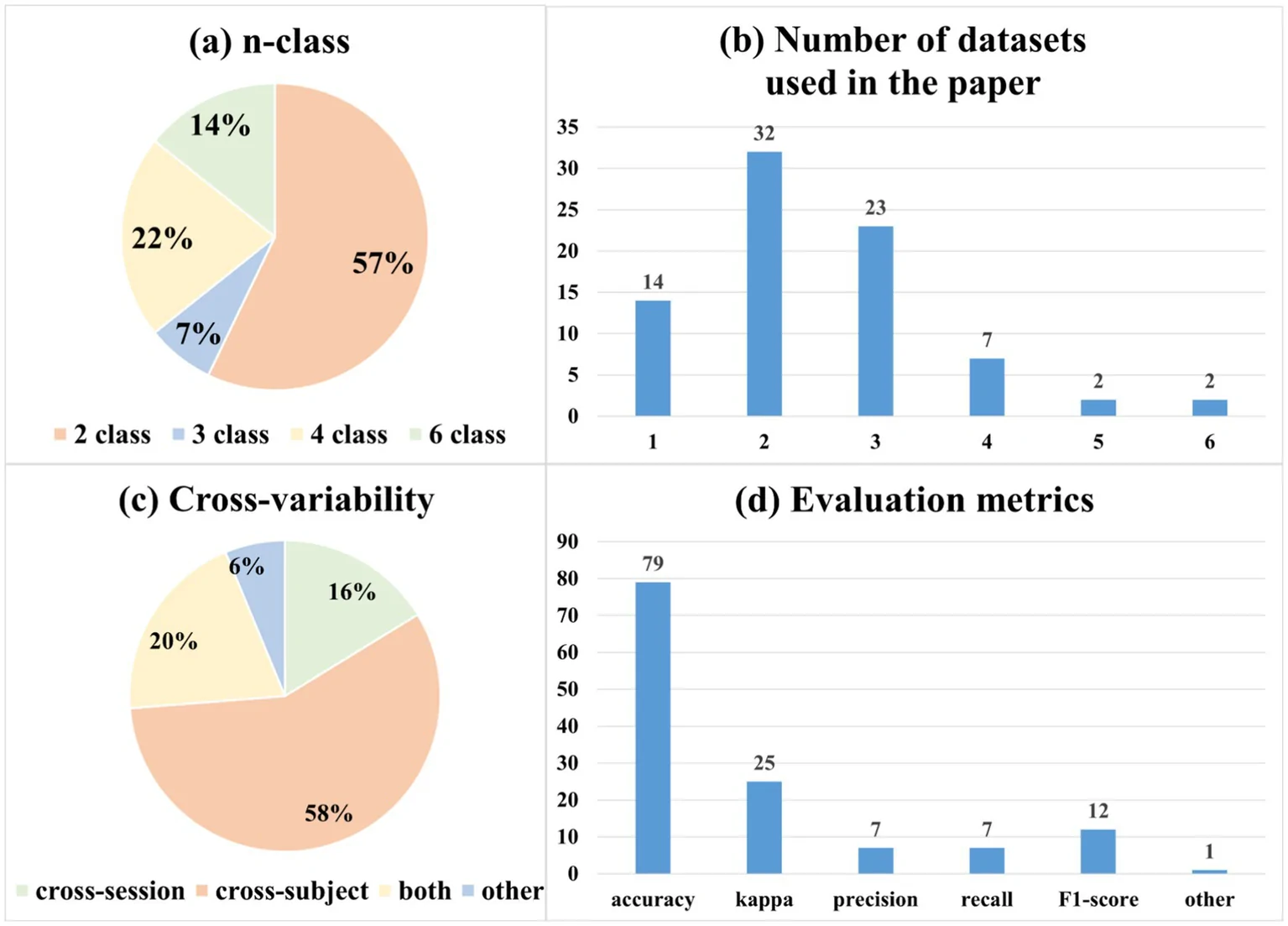
Preliminary analyses. **(a)** datasets class distribution, **(b)** the number of datasets used per paper, **(c)** cross-variability study types, and **(d)** evaluation metrics applied.

The preliminary analysis of 80 papers is displayed in Figure 6. As shown in Figure 6a, binary classification datasets were more prevalent (57%). Next came four-classification datasets (22%), while three-classification and six-classification datasets were less common, accounting for 7 and 14%, respectively. The majority of these datasets focused on left- or right-hand MI intention. Furthermore, binary MI EEG decoding demonstrated lower variability, resulting in reduced complexity. As shown in Figure 6b, among papers analyzing cross-variability in MI EEG signals, 83% employ more than two distinct datasets to validate model performance. Integrating multiple datasets not only effectively validates the model’s capability in handling cross-variability but also enhances the persuasiveness of its generalization performance. In variability studies, as shown in Figure 6c, among the 80 included studies, 46 studies focused exclusively on cross-subject analysis, while 13 studies investigated only cross-session variability. In addition, 16 studies simultaneously considered both cross-subject and cross-session variations. Beyond these two major variability scenarios, 5 studies further explored other types of cross-variability, including 4 cross-dataset investigations and 1 cross-task analysis.

Regarding model evaluation metrics, as shown in Figure 6d, most papers use accuracy as the primary evaluation metric, while utilizing kappa coefficient, F1-score, precision, and recall as supplementary performance measures. Accuracy stands as one of the most intuitive and interpretable evaluation metrics, providing a direct measurement of a model’s prediction correctness across the entire dataset. The kappa coefficient, by accounting for random agreement in classification outcomes, offers a more objective performance evaluation, particularly valuable for imbalanced class distributions. By measuring the ratio of true positives to all predicted positives, precision indicates the model’s strictness and exactness. Recall measures the ratio of correctly identified positives, complementing precision for evaluating positive-class recognition capability. By consolidating recall and precision into a single harmonic mean, the F1-score generates a unified view of positive-class classification performance.

## 3 Deep learning-based feature representation learning

We categorize existing deep learning-based methods according to their core feature learning mechanisms. Specifically, the methods are grouped into four categories: local spatiotemporal feature modeling, multi-branch feature fusion, global context modeling, and sequence representation learning. Furthermore, we analyze their applicable scenarios and potential advantages by considering the targeted cross-variability sources and target-domain data availability. Table 2 provides a concise overview of these models.

**Table 2:** Summary of deep learning models.

Core mechanism/ learning objective	Paper	Sources of variability	Availability of target data	Deployment constraints
Local spatiotemporal modeling	Olvera et al. (2024)	cross-subject	No target data	Calibration-free High computational load
	Khabti et al. (2024)	cross-subject	No target data	Calibration-free Computationally lightweight
	Li et al. (2024a)	cross-subject	No target data	Calibration-free High computational load
	Sun et al. (2021)	cross-session	No target data	Computationally lightweight
	Autthasan et al. (2022)	cross-session	Fully labeled calibration data	Calibration-free Real-time streaming processing
		cross-subject	No target data	
	Saideepthi et al. (2023)	cross-session	Fully labeled calibration data	Calibration-free Computationally lightweight
		cross-subject	No target data	
	Wu et al. (2024)	cross-session	Fully labeled calibration data	Calibration-free Real-time streaming processing
		cross-subject	No target data	
	Wang et al. (2025c)	cross-session	Fully labeled calibration data	Calibration-free Computationally lightweight
		cross-subject	No target data	
	Xu et al. (2020)	cross-subject	Fully labeled calibration data	Calibration-free
		cross-dataset	No target data	
Multi-branch feature fusion	Liu et al. (2021)	cross-subject	No target data	High computational load
	Chowdhury et al. (2023)	cross-subject	Fully labeled calibration data	High computational load
	Dong et al. (2023)	cross-subject	No target data	Calibration-free Computationally lightweight
	Jin et al. (2025)	cross-subject	No target data	Calibration-free Computationally lightweight
	Jia et al. (2023)	cross-session	Fully labeled calibration data	High computational load
	Yu et al. (2024)	cross-session	Fully labeled calibration data	High computational load
Global context modeling	Deny et al. (2023)	cross-subject	No target data	High computational load
	Luo et al. (2023)	cross-subject	No target data	Calibration-free Computationally lightweight
	Sun et al. (2024)	cross-subject	No target data	Calibration-free High computational load
	Altaheri et al. (2023a)	cross-session	Fully labeled calibration data	Calibration-free Edge-deployable
		cross-subject	No target data	
	Altaheri et al. (2023a)	cross-session	Fully labeled calibration data	Computationally lightweight
		cross-subject	No target data	
State-space sequence modeling	Wang et al. (2025b)	cross-subject	No target data	Calibration-free Computationally lightweight
		cross-dataset	No target data	

### 3.1 Local spatiotemporal modeling

Local spatiotemporal modeling methods learn spatial distributions and temporal dynamics of EEG signals to automatically extract discriminative representations, thereby reducing the dependence on handcrafted feature design. In cross-subject scenarios, these methods typically employ local feature extraction mechanisms, such as convolutional structures, to learn robust EEG representations and improve model generalization. Khabti et al. (2024) introduced an optimal channel selection method for multi-class MI EEG classification based on a Fusion CNN with an Attention Block (FCNNA). The method selects informative EEG channels and enhances the extraction of discriminative features. Olvera et al. (2024) explored a quantum computing-assisted EEG classification approach. They applied a quantum genetic algorithm for feature selection and combined it with a hybrid classical-quantum neural network to enhance feature representation, providing a new feature learning strategy for cross-subject EEG decoding. In addition, Li et al. (2024a) proposed a self-supervised contrastive learning framework to decode MI EEG, which integrates CNN and attention mechanism to enhance network robustness.

Based on the nonstationary and time-varying characteristics of EEG signals, research (Sun et al., 2021) developed an SSD-SE CNN framework, short for Sparse Spectrotemporal Decomposition Squeeze-and-Excitation. In cross-session scenarios, it learns more stable spatiotemporal features, providing a local feature modeling strategy to alleviate performance degradation caused by temporal variations in EEG signals.

For cross-subject and cross-session variations, existing studies mainly focus on learning stable and discriminative EEG representations. Local spatiotemporal modeling methods extract robust features through mechanisms such as convolution and multi-task learning to alleviate the effects of inter-subject differences and temporal non-stationarity. Autthasan et al. (2022) proposed an end-to-end multi-task learning model termed MIN2Net, which combines a multi-task autoencoder with deep metric learning to learn compact and discriminative EEG representations. Saideepthi et al. (2023) introduced a Longest Consecutive Repetition (LCR) post-processing strategy based on EEGNet to improve the stability of sliding-window predictions. Wu et al. (2024) proposed a Sliding Window-Based Prescreening and Classification (SWPC) method for MI EEG classification in asynchronous BCIs. Furthermore, Wang et al. (2025b) proposed a Dual-Branch Convolutional Transformer network (DBConformer). It adopts parallel temporal and spatial Conformers with a lightweight channel attention module.

In addition to cross-session and cross-subject experimental paradigms, some researchers have verified the generalization ability of models through cross-dataset experimental methods. Evaluating deep learning models on eight MI EEG datasets, Xu et al. (2020) found cross-dataset variability harms generalization. They put forward an online pre-alignment method via recursive Riemannian mean optimization. This method boosted cross-dataset generalization without extra calibration data.

### 3.2 Multi-branch feature fusion

Multi-branch feature fusion methods construct multiple parallel feature learning pathways to extract complementary information from different scales, modalities, or feature spaces, thereby improving the completeness and robustness of EEG representations. In cross-subject scenarios, significant differences in spatial patterns and signal distributions across individuals make it difficult for a single feature extraction pathway to learn sufficiently generalizable representations.

Liu et al. (2021) integrated raw EEG signals with handcrafted features and proposed a CNN-based multi-task learning model, Multiscale Space–time-frequency feature-guided Multitask Learning (MSML). The method jointly learns multi-scale spatiotemporal-frequency features to improve cross-subject classification performance. Chowdhury et al. (2023) proposed a five-branch two-dimensional CNN architecture, where multiple branches learn different feature representations to enhance MI EEG cross-subject decoding capability. Dong et al. (2023) proposed a novel dual-branch MultiScale AutoEncoder Network (MSAENet), which integrates spatial-spectral feature preprocessing with multi-scale convolution, and introduces a center loss function to enhance intra-class compactness. Jin et al. (2025) proposed a Multiscale Spatial–Temporal Feature Fusion Network (MSTFNet), which further integrates multi-scale spatiotemporal features, channel attention, and regularization strategies to improve the modeling capability for complex EEG patterns.

In cross-session scenarios, EEG signals often exhibit temporal drift and non-stationarity. Therefore, multi-branch structures have been further explored to integrate spatiotemporal or spectral features at different scales and learn more stable EEG representations.

Jia et al. (2023) proposed a Multi-Branch Spectral-Temporal CNN with Efficient Channel Attention and Light Gradient Boosting Machine (MBSTCNN-EL), which augments critical channels via an efficient channel attention module. Yu et al. (2024) proposed a hybrid architecture integrating deep learning and machine learning models, termed the Multi-Branch CNN with Temporal Convolutional Network (MBCNN-TCN), which integrates a multi-branch convolutional network with a temporal convolutional network to jointly model spatial and temporal features and improve cross-session decoding performance.

### 3.3 Global context modeling

Unlike local feature extraction methods, global context modeling approaches utilize Transformer and self-attention mechanisms to capture long-range temporal dependencies in EEG signals and learn high-level global representations. In cross-subject scenarios, significant variations in EEG patterns across individuals pose challenges for model generalization. These methods improve robustness to inter-subject variations by enhancing the modeling of informative temporal segments and global feature relationships. Deny et al. (2023) proposed a hierarchical Transformer (HT) framework. The method uses a low-level Transformer to encode short-term segment features and a high-level Transformer to further model global dependencies among different temporal segments. Luo et al. (2023) proposed the Shallow Mirror Transformer (SMT), which combines a shallow Transformer with mirror data augmentation and ensemble learning strategies. By expanding the training data distribution, SMT improves cross-subject generalization capability. Sun et al. (2024) outlined a temporal dependency learning framework for MI using Contrastive Learning and Self-Attention (CLS), which can capture EEG temporal dependencies while extracting cross-subject invariant features.

To address EEG distribution variations in cross-subject and cross-session scenarios, Altaheri et al. (2023a) developed an Attention-Based Temporal Convolutional Network (ATCNet) for MI EEG decoding. This model employs multiple techniques to enhance classification performance with relatively few parameters. Building on this foundation, researchers further introduced dynamic convolution and multi-head attention, proposing the Dynamic Attention Temporal Convolution Network (D-ATCNet) to enhance MI EEG classification results using few parameters (Altaheri et al., 2023b).

### 3.4 Global context modeling

With the development of foundation models, long-sequence modeling methods have gradually been applied to EEG analysis. These methods learn general representations from large-scale datasets and improve model adaptability to variations across subjects and datasets. Compared with traditional Transformer architectures, state-space model-based approaches can capture long-range dependencies with lower computational complexity, providing a new direction for EEG representation learning across different data distributions. EEGMamba, a novel EEG foundation model proposed by Wang et al. (2025c), learns universal representations for multiple BCI tasks through a bidirectional Mamba backbone and patch-masked reconstruction pretraining, effectively integrating spatio-temporal-spectral features.

## 4 Transfer learning-based adaptation strategies

Unlike deep learning-based methods, transfer learning-based approaches mainly achieve cross-domain adaptation by reducing discrepancies between source and target domains or leveraging previously learned knowledge (Wu et al., 2021). To systematically analyze the roles of different transfer mechanisms in addressing cross-variability issues, we categorize existing methods according to their core adaptation strategies, including statistical distribution alignment, parameter transfer/fine-tuning, representation disentanglement learning, meta-learning, data augmentation, contrastive learning, and adversarial learning. Furthermore, we discuss the characteristics of different strategies in cross-variability scenarios by considering target data availability, feature alignment spaces, and deployment conditions. Table 3 presents a summary of the models.

**Table 3:** Summary of transfer learning models.

Core mechanism/learning objective	Paper	Sources of variability	Availability of target data	Alignment space	Deployment constraints
Statistical distribution alignment	Zhang and Wu (2020)	Cross-subject	Unlabeled target data	Riemannian SPD manifold	Unsupervised subspace alignment
	Zhao et al. (2020)	Cross-subject	Unlabeled target data	Deep feature representation space	Unsupervised offline alignment
	Zhu et al. (2021)	Cross-subject	Unlabeled target data	Deep feature representation space	Unsupervised offline alignment
	Zhang et al. (2021a)	Cross-subject	Unlabeled target data	Raw EEG signal space	Unsupervised offline alignment
	Xu et al. (2021)	Cross-subject	Fully labeled calibration data	Riemannian SPD manifold	Supervised centralized training
	Wan et al. (2022)	Cross-subject	Fully labeled calibration data	Raw EEG signal space	Unsupervised offline alignment
	Gaur et al. (2022)	Cross-subject	No target data	Riemannian SPD manifold	Zero−/few-shot inference
	Chen et al. (2022a)	Cross-subject	Unlabeled target data	Deep feature representation space	Unsupervised offline alignment
	Chen et al. (2022b)	Cross-subject	Unlabeled target data	Deep feature representation space	Unsupervised offline alignment
	Zhang et al. (2022)	Cross-subject	Unlabeled target data	Tangent space + Feature subspace	Zero−/few-shot inference
	Luo (2023)	Cross-subject	Unlabeled target data	Deep feature representation space	Unsupervised offline alignment
	Xu et al. (2024b)	Cross-subject	Unlabeled target data	Riemannian SPD manifold	Unsupervised subspace alignment
	Luo (2024)	Cross-subject	Unlabeled target data	Deep feature representation space	Unsupervised offline alignment
	Li et al. (2025)	Cross-subject	Unlabeled target data	Riemannian SPD manifold	Unsupervised offline alignment
	Yang et al. (2025a)	Cross-subject	Unlabeled target data	Reproducing Kernel Hilbert Space	Unsupervised offline alignment
	Peterson et al. (2022)	Cross-session	Fully labeled calibration data	Deep feature representation space	Supervised online adaptation
	Pan et al. (2023)	Cross-session	Few labeled target data	Riemannian SPD manifold	Few-shot offline calibration
	Zhang et al. (2023a)	Cross-session	Unlabeled target data	Deep feature representation space	Unsupervised offline alignment
	Zhang et al. (2023d)	Cross-session	Fully labeled calibration data	Deep feature representation space	Supervised centralized training
	Liu et al. (2024)	Cross-session	Unlabeled target data	Riemannian SPD manifold	Unsupervised offline alignment
	Zhong et al. (2023)	Cross-subject Cross-session	Unlabeled target data	Deep feature representation space	Unsupervised offline alignment
	Tang et al. (2023)	Cross-subject Cross-session	Unlabeled target data	Riemannian SPD manifold	Unsupervised offline alignment
	Xu et al. (2024a)	Cross-subject Cross-session	Unlabeled target data	Raw EEG signal space	Unsupervised offline alignment
	Zhuo et al. (2024)	Cross-subject Cross-session	Unlabeled target data	Riemannian SPD manifold	Unsupervised offline alignment
	Han et al. (2023)	Cross-subject Cross-dataset	Few labeled target data	Deep feature representation space	Supervised centralized training
Parameter transfer/fine-tuning	Zhang et al. (2022)	Cross-subject	Unlabeled target data	Deep feature representation space	Zero−/few-shot inference
	Mammone et al. (2023)	Cross-subject	Fully labeled calibration data	Deep feature representation space	Supervised few-shot fine-tuning
	Li et al. (2023)	Cross-subject	Few labeled target data	Deep feature representation space	Supervised few-shot fine-tuning
	Wang et al. (2024a)	Cross-subject	Unlabeled target data	Deep feature representation space	Unsupervised offline alignment
	Li et al. (2024c)	Cross-subject	Few labeled target data	Riemannian SPD manifold	Supervised few-shot fine-tuning
	Lee et al. (2024b)	Cross-subject	No target data	Deep feature representation space	Zero−/few-shot inference
	Nagarajan et al. (2024)	Cross-subject	Fully labeled calibration data	Deep feature representation space	Supervised offline adaptation
	Zhang et al. (2021b)	Cross-subject Cross-session	Fully labeled calibration data	Deep feature representation space	Supervised few-shot fine-tuning
	Xia et al. (2022)	Cross-subject Cross-session	Unlabeled target data	Tangent space + Feature subspace	Zero−/few-shot inference
	Roy (2022)	Cross-subject Cross-session	Fully labeled calibration data	Deep feature representation space	Supervised few-shot fine-tuning
	Liang et al. (2024)	Cross-subject Cross-session	Few labeled target data	Deep feature representation space	Supervised few-shot fine-tuning
	Miao et al. (2023)	Cross-subject Cross-task	Few labeled target data	Raw EEG signal space	Supervised few-shot fine-tuning
Representation disentanglement learning	Sartipi and Cetin (2024)	Cross-subject	No target data	Deep feature representation space	Zero−/few-shot inference
	Zhi et al. (2025)	Cross-subject	No target data	Deep feature representation space	Zero-shot inference deployment
	Jeon et al. (2023)	Cross-subject Cross-session	Fully labeled calibration data	Deep feature representation space	Supervised centralized training
Meta-learning	Ng and Guan (2024)	Cross-subject	No target data	Deep feature representation space	Zero−/few-shot inference
	An et al. (2024)	Cross-subject Cross-dataset	Few labeled target data	Deep feature representation space	Zero−/few-shot inference
Data augmentation	Zhang et al. (2020)	Cross-subject	Fully labeled calibration data	Graph structure space	Supervised centralized training
	Feng et al. (2022)	Cross-subject	Fully labeled calibration data	Reproducing Kernel Hilbert Space	Supervised centralized training
	Bi and Chu (2023)	Cross-subject	Fully labeled calibration data	Deep feature representation space	Supervised centralized training
	Zhang et al. (2023c)	Cross-subject	Few labeled target data	Graph structure space	Supervised few-shot fine-tuning
	Tao et al. (2024)	Cross-session	Fully labeled calibration data	NR	Supervised centralized training
Contrastive learning	Zhu et al. (2024)	Cross-subject	Unlabeled target data	Deep feature representation space	Zero−/few-shot inference
	Xu et al. (2025)	Cross-subject	Unlabeled target data	Deep feature representation space	Unsupervised offline alignment
	Asgarian et al. (2024)	Cross-session	Unlabeled target data	Deep feature representation space	Unsupervised offline alignment
Adversarial learning	Song et al. (2023)	Cross-subject	Fully labeled calibration data	Deep feature representation space	Supervised centralized training
	Yin et al. (2024)	Cross-subject	Unlabeled target data	Raw EEG signal + Feature subspace	Unsupervised offline alignment
	Lee et al. (2024a)	Cross-subject	Unlabeled target data	Deep feature representation space	Unsupervised offline alignment
	Zhang et al. (2024a)	Cross-subject	Unlabeled target data	Deep feature representation space	Unsupervised offline alignment
	Zhao et al. (2021)	Cross-session	Fully labeled calibration data	Deep feature representation space	Supervised centralized training
	Zhang et al. (2024c)	Cross-session	Unlabeled target data	Deep feature representation space	Unsupervised offline alignment
	Chen et al. (2024)	Cross-session	Fully labeled calibration data	Raw EEG signal space	Supervised centralized training
	She et al. (2023)	Cross-subject Cross-session	Unlabeled target data	Deep feature representation space	Unsupervised offline alignment
	Li et al. (2024b)	Cross-subject Cross-session	Unlabeled target data	Graph signal space	Unsupervised offline alignment

### 4.1 Statistical distribution alignment

Statistical distribution alignment methods mainly reduce distribution discrepancies across subjects to learn EEG representations with improved cross-subject generalization ability. Due to individual variability, the feature distributions of EEG signals often differ across subjects, resulting in substantial distribution shifts between source and target domains. Therefore, existing studies employ strategies such as distribution matching and feature-space alignment to mitigate inter-subject variations and improve model adaptation to unseen subjects.

Distribution matching-based domain adaptation methods achieve cross-subject knowledge transfer by explicitly modeling discrepancies in marginal or joint distributions between source and target domains. Zhang and Wu (2020) proposed the Manifold Embedded Knowledge Transfer (MEKT) method, which incorporates a domain transferability estimation strategy to evaluate the transferability of different source domains in the manifold space. The method supports both single-source and multi-source knowledge transfer. Zhao et al. (2020) modeled joint distribution discrepancies by considering both marginal distribution shifts and class-related distribution variations, enabling more fine-grained domain alignment. Chen et al. (2022a) combined joint distribution adaptation with optimal transport to reduce joint probability discrepancies between source and target domains by integrating feature and label information. Furthermore, Chen et al. (2022b) and Xu et al. (2024b) further optimized the distribution transfer process from the perspectives of multi-subdomain adaptation and local structure preservation, respectively.

Geometry-based alignment methods exploit the non-Euclidean structure of EEG covariance features to learn more stable cross-subject representations in the Riemannian manifold space. Zhang et al. (2021a) proposed the Sub-Band Target Alignment Common Spatial Pattern (SBTACSP) method, which enhances the transferability of spatial filtering features through target-domain alignment. Xu et al., 2021 and Gaur et al. (2022) employed Riemannian manifold mapping and tangent space representation to transform covariance matrices into feature spaces suitable for transfer learning. Li et al. (2025) further integrated Symmetric Positive Definite (SPD) manifold alignment with low-rank reconstruction. The method improves the robustness of cross-subject MI EEG decoding through multi-branch feature fusion.

Sample selection and adaptive transfer methods mainly focus on improving the utilization of target-domain information. These methods reduce uncertainty in cross-subject transfer by selecting reliable samples or constructing auxiliary domains. Zhu et al. (2021) proposed the Multi-source Fusion Adaptation Regularization (MFAR) method, which alleviates the impact of individual differences caused by limited training samples through multi-source fusion and regularization strategies. Wan et al. (2022) adopted a temporal-segment-based data alignment strategy to improve transfer stability under degraded data conditions. Zhang et al. (2022) achieved source-free knowledge transfer by constructing a virtual intermediate domain, thereby reducing distribution discrepancies between source and target domains. Luo (2024) proposed the Dual Regularized Spatial–Temporal Features Adaptation (DRSTFA) method, which improves the learning of transferable features in cross-subject scenarios through multi-source domain sample selection and spatiotemporal feature adaptation. Luo et al. (2023) and Yang et al. (2025a) further incorporated discriminative feature selection and high-confidence pseudo-label filtering to improve the reliability of target-domain adaptation.

In cross-session decoding, statistical distribution alignment methods typically alleviate session-related distribution shifts through alignment strategies in feature space, probability distribution space, or Riemannian manifold space. Peterson et al. (2022) adopted the optimal transport approach to adjust the relationship between target-domain samples and the source-domain classifier, achieving cross-domain distribution matching. Pan et al. (2023) proposed the Riemannian geometry-based Adaptive Boosting and Voting Ensemble (RAVE) method, which combines Riemannian geometric representations with ensemble learning to enhance feature consistency in geometric space for cross-session adaptation. Zhang et al. (2023a) proposed the Siamese Deep Domain Adaptation (SDDA) framework, which integrates Maximum Mean Discrepancy (MMD) constraints with discriminative feature learning. MI-CAT (Zhang et al., 2023b) and RGDDANet (Liu et al., 2024) further explored deep domain adaptation methods based on attention mechanisms and Riemannian manifold learning. These methods perform feature alignment in latent representation spaces to reduce session-related discrepancies.

Due to significant feature shifts across subjects and sessions, statistical distribution alignment methods typically learn more stable domain-invariant representations through strategies such as covariance matching, statistical feature alignment, and Riemannian space mapping. Zhong et al. (2023) proposed the Deep Domain Adaptation Framework with Correlation Alignment (DDAF-CORAL), which reduces statistical distribution discrepancies between domains by aligning feature covariances of source and target domains. Tang et al. (2023) proposed the Rotation Alignment Domain Adaptation method with Riemannian mean (RMRA), which uses rotation alignment in the Riemannian space to match covariance structures between source and target domains. Xu et al. (2024a) proposed the Feature Relation Contrastive Network (FRCN), which combines covariance alignment with source-domain selection. By selecting data with higher similarity to the target domain, FRCN improves the effectiveness of multi-source transfer. Zhuo et al. (2024) further optimized the domain adaptation process in manifold space based on the Log-Euclidean Metric (LEM) and Riemannian Procrustes analysis.

In cross-subject and cross-dataset scenarios, differences in acquisition devices, EEG channel layouts, and subjects can lead to significant domain shifts. Han et al. (2023) proposed a cross-dataset adaptation framework that integrates graph neural networks and transfer learning. This approach effectively aggregates knowledge from multiple datasets with varying electrode layouts, enhancing the generalization ability for cross-subject and datasets MI EEG classification.

### 4.2 Parameter transfer/fine-tuning

Parameter transfer and fine-tuning methods enable rapid adaptation to new subjects by reusing source-domain model parameters, feature representations, or classification knowledge. In cross-subject MI EEG decoding, these methods typically learn general representations from multi-subject data and reduce the impact of individual differences through parameter adjustment, source-model selection, or auxiliary-task transfer.

Zhang et al. (2022) proposed the Multi-Source Decentralized Transfer (MSDT) method, which transfers source-model parameters or prediction outputs and dynamically adjusts the weights of different source models. This approach enables cross-subject knowledge transfer without sharing raw data while considering privacy protection requirements. Mammone et al. (2023) proposed the Autoencoder-Filter Bank Common Spatial Patterns (AE-FBCSP), which combines global and subject-specific feature representations. Li et al. (2023) proposed the Multi-Direction Transfer Learning (MDTL) strategy, which dynamically selects source domains and progressively transfers feature extraction parameters. Wang et al. (2024a) proposed an Iterative Self-training Multisubject Domain Adaptation (ISMDA), which integrates an iterative self-training strategy to optimize target-domain adaptation and reduce the influence of source-domain bias on transfer performance.

Furthermore, several studies have explored the application of parameter transfer in more complex scenarios. Li et al. (2024c) combined Riemannian coordinate alignment with a Softmax parameter transfer strategy to reduce the risk of negative transfer by optimizing the parameter adaptation process. Lee et al. (2024b) proposed the Multi-Task Heterogeneous Ensemble Learning (MHEL) framework, which integrates multi-task learning and heterogeneous ensemble strategies to improve cross-subject MI EEG decoding in clinical populations. Nagarajan et al. (2024) further investigated cross-population transfer from healthy subjects to stroke patients. By leveraging data from healthy populations, this method alleviates the limited availability of clinical data and provides a new direction for EEG knowledge transfer across different populations.

In cross-subject and cross-session scenarios, these methods typically adjust partial network parameters or transfer knowledge from source models to reduce the need for retraining and improve adaptation to new domains. Zhang et al. (2021b) proposed the Deep CNN-based Adaptive Transfer Learning (DCATL) framework, which adjusts pre-trained model parameters through multiple fine-tuning strategies. Xia et al. (2022) proposed the Augmentation-based Source-Free Adaptation (ASFA) method. This approach enables target-domain adaptation while preserving source-domain privacy and provides a strategy for cross-session transfer without source data sharing. Roy (2022) investigated the effects of different parameter updating ranges on transfer performance through multi-scale CNN feature fusion and adaptive fine-tuning strategies. Liang et al. (2024) further developed an end-to-end decoding framework by integrating multi-scale feature learning and attention mechanisms. The framework improves cross-domain EEG feature transferability by combining local and global representations.

To explore the feasibility of cross-task transfer learning (from ME to MI), Miao et al. (2023) proposed an Explainable Cross-Task Adaptive Transfer Learning (ExCTATL) method. This approach leverages extensively pre-trained models based on ME EEG data to enhance the classification of MI EEG data.

### 4.3 Representation disentanglement learning

Representation disentanglement learning separates domain-related variations from task-relevant information in EEG signals to learn more stable domain-invariant representations. In cross-subject MI EEG decoding, substantial subject-specific differences exist across individuals. These methods enhance class-related features while suppressing subject-related information to improve generalization to unseen subjects. Sartipi and Cetin (2024) proposed a semi-supervised deep decoding framework that learns transferable representations using a small amount of labeled target-domain data, reducing the dependence on large-scale labeled data. Zhi et al. (2025) proposed the Supervised Contrastive Learning Domain Generalization Network (SCLDGN), which combines domain alignment with class-discriminative constraints. The method learns domain-invariant and discriminative representations within a domain generalization framework, improving adaptation to cross-subject MI EEG and ME EEG tasks.

Since session variations often introduce task-irrelevant signal fluctuations, representation disentanglement learning improves robustness to session changes by suppressing irrelevant features and enhancing discriminative information. Jeon et al. (2023) proposed a mutual information-based deep representation learning method that decomposes features into class-related and class-independent components to reduce the impact of negative transfer. The method further enhances class discriminability by introducing mutual information constraints between local and global representations, thereby improving the generalization capability of cross-session EEG decoding.

### 4.4 Meta-learning

Meta-learning facilitates models to swiftly adjust to new tasks by leveraging multi-task training with limited data, effectively addressing EEG data scarcity problem. An et al. (2024) suggested a Dual-Attention Relation Network (DA-RelationNet). They combined a dual-attention mechanism with meta-learning and designed a fine-tuning strategy without additional data. Ng and Guan (2024) put forward a cross-subject meta-learning framework, which optimizes model parameters using a meta-loss function, enabling model to quickly adapt to new subjects requiring a small amount of target data.

### 4.5 Data augmentation

Data augmentation methods improve robustness to cross-subject variations by expanding source-domain distributions or constructing more transferable training samples. In cross-subject MI EEG decoding, substantial distribution differences exist across subjects. Therefore, these methods typically incorporate sample transfer, data reconstruction, or auxiliary learning strategies to increase training diversity and reduce dependence on subject-specific features. Zhang et al. (2020) combined a convolutional neural network with the Instance Transfer Subject-Independent (ITSD) strategy to enhance cross-subject MI EEG classification through instance-level transfer. Feng et al. (2022) proposed an adaptive cross-subject transfer framework that integrates Kernel Mean Matching (KMM) and Transfer Learning Adaptive Boosting (TrAdaBoost). The framework reduces negative transfer by adjusting source-domain sample weights and improves model generalization. Bi and Chu (2023) proposed the Transfer Data Learning Network (TDLNet), which combines transfer data learning with attention mechanisms to enhance cross-subject recognition in multi-class motor imagery tasks. Zhang et al. (2023b) proposed Co-Teaching Graph Learning (CTGL), which alleviates individual differences through a collaborative learning mechanism and improves adaptation to data from different subjects.

In cross-session MI EEG decoding, signal fluctuations and feature distribution changes across recording sessions can reduce model robustness. Data augmentation methods alleviate this issue by increasing feature diversity and improving representation stability, thereby reducing dependence on session-specific patterns. Tao et al. (2024) proposed the Attention-Based Dual-Scale Fusion CNN (ADFCNN), which enhances multi-scale feature utilization through dual-scale spatiotemporal feature extraction and attention-based fusion. The method improves model adaptation to spatiotemporal variations in cross-session scenarios.

### 4.6 Contrastive learning

Contrastive learning methods learn domain-invariant EEG representations by constraining sample relationships across domains. In cross-subject MI EEG decoding, these methods reduce subject-related variations by enhancing class consistency and feature discrimination. Zhu et al. (2024) proposed a privacy-preserving source-free adaptation framework that combines contrastive losses to achieve cross-subject knowledge transfer without source-domain data. Xu et al. (2025) proposed Contrastive Learning-based Unsupervised Multi-source Domain Adaptation (CLUDA), which aligns multi-source and target-domain representations through contrastive learning and reduces source-domain discrepancies to learn stable cross-subject EEG representations.

In cross-session MI EEG decoding, temporal and spatial feature shifts across recording sessions can affect model robustness. Contrastive learning methods improve adaptation to session variations by enhancing representation consistency and integrating multi-view information. Asgarian et al. (2024) proposed the Multiview Adversarial Contrastive Network (MACNet), which integrates multi-view EEG representations from Euclidean and Riemannian spaces. By using contrastive learning to enhance consistency among different representations, MACNet improves adaptation to spatiotemporal variations across sessions.

### 4.7 Adversarial learning

Adversarial learning introduces domain discrimination mechanisms to learn domain-invariant features that are difficult to distinguish between source and target domains, thereby reducing distribution discrepancies across domains. In cross-subject MI EEG decoding, substantial inter-subject variations exist, and adversarial learning methods typically incorporate domain-adversarial training, multi-source domain selection, or generative transfer strategies to improve adaptation to unseen subjects. Song et al. (2023) proposed Global Adaptive Transformer (GAT), which integrates attention mechanisms with adversarial learning to enhance cross-subject feature alignment. Yin et al. (2024) proposed Generative Inter-Subject Transfer Generative Adversarial Networks (GITGAN), which uses generative adaptation and source selection to improve unsupervised transfer. Lee et al. (2024a) further emphasized source-domain selection to reduce negative transfer. Zhang et al. (2024a) proposed Prototype-Supervised Adversarial Transfer learning (PSAT), which introduces prototype supervision to enhance domain alignment and cross-subject generalization.

In cross-session scenarios, EEG signals are affected by temporal variations and experimental conditions. Adversarial learning methods typically improve feature consistency between source and target domains through global or local domain alignment, enhancing adaptation to session variations. Zhao et al. (2021) proposed Deep Representation Domain Adaptation (DRDA), which aligns domain distributions through adversarial learning to learn domain-invariant representations. Zhang et al. (2024c) combined global and local alignment strategies to improve adaptation under unsupervised and semi-supervised settings. Chen et al. (2024) proposed Alignment-Based Adversarial Training (ABAT), which introduces session-level alignment before adversarial training to balance robustness and decoding performance.

In cross-subject and cross-session scenarios, individual differences and temporal variations lead to EEG feature shifts. Adversarial learning methods improve alignment stability and transferability by optimizing adversarial objectives or incorporating structural prior information. She et al. (2023) proposed a Wasserstein distance-based enhanced domain adaptation network, which optimizes distribution matching between source and target domains to alleviate gradient vanishing in traditional adversarial losses. Li et al. (2024b) further introduced a neuroanatomical prior-guided subdomain adversarial network (SAN) by incorporating EEG electrode spatial information. The method learns domain-shared features and reduces interference from redundant information.

## 5 Discussion

### 5.1 Findings

In MI EEG cross-variability research, public datasets serve as standardized benchmarks for model evaluation. These datasets not only provide standardized data formats and annotations but also ensure reproducibility and comparability across different models. These datasets feature heterogeneous subject cohorts and experimental protocols that rigorously assess generalization against inter-subject differences and session-dependent drift.

The datasets exhibit two key characteristics: First, by incorporating diverse subject cohorts and differentiated experimental protocols, they enable systematic evaluation of model generalization capabilities against inter-subject variability and cross-session signal drift. Second, established multi-session benchmark datasets (e.g., BCI IV 2a: 9 subjects × 2 sessions; BCI IV 2b: 9 subjects × 5 sessions) comprehensively capture intra-subject signal variations over time, providing an ideal platform for both cross-subject and cross-session classification (Autthasan et al., 2022; Altaheri et al., 2023a; Han et al., 2023; Li et al., 2024b). Consequently, these datasets have become indispensable resources for assessing the capability of neuroadaptive models to handle variability in MI EEG research.

#### 5.1.1 Deep learning models

Analysis of prevailing deep learning models reveals that most innovations build upon CNN or Transformer architectures. CNNs leverage convolution operations to intrinsically align with the spatiotemporal structure of EEG signals, enabling efficient extraction of localized spectral-spatial features fundamental to EEG pattern recognition (Sun et al., 2021; Altaheri et al., 2023b; Saideepthi et al., 2023; Li et al., 2024a). In contrast, transformers employ self-attention mechanisms to dynamically weight critical temporal segments or channels, effectively mitigating temporal signal drift induced by cross-variability (Dong et al., 2023; Sun et al., 2024). Advanced model iterations further integrate attention mechanisms, dynamic convolution kernels, and distribution alignment strategies. Fundamentally, these architectural enhancements bolster robustness against EEG nonstationary, inter-subject variability, and environmental noise, thereby advancing performance in cross-subject decoding tasks.

#### 5.1.2 Transfer learning model

Contemporary transfer learning models primarily innovate through domain adaptation or multi-source selection paradigms. Domain adaptation techniques reduce inter-domain discrepancies through feature distribution alignment, effectively mitigating cross-subject EEG distribution shifts (Jeon et al., 2023; Zhong et al., 2023; Zhang et al., 2024c). Multi-source selection strategies alleviate domain divergence by implementing similarity-based source filtering mechanisms (Yin et al., 2024; Xu et al., 2024a). State-of-the-art innovations integrate advanced techniques, including semi-supervised learning and graph-structured transfer learning (Zhang et al., 2023b; Sartipi and Cetin, 2024). These advancements inherently enhance EEG models’ robustness against inter-subject variability through transfer optimization, leading to significant gains in cross-domain decoding.

#### 5.1.3 Performance summaries on public datasets

We further summarize the experimental settings and results of cross-subject and cross-session MI-EEG decoding studies based on the four-class BCI IV 2a dataset, as presented in Tables 4, 5. These tables summarize the preprocessing methods, train/test split protocols, target-label availability, calibration data usage, validation procedure, baseline configurations, hyperparameter selection, reported classification performance, and uncertainty statistics across studies. It should be noted that Tables 4, 5 provide a descriptive summary of methodological characteristics and experimental settings rather than direct performance rankings or comparisons. Further analysis of the experimental settings in Tables 4, 5 reveals substantial differences among studies in data preprocessing and split protocols. Some studies employed different filtering bands and normalization methods for preprocessing. The data partition strategies mainly included fixed splits, cross-validation, and leave-one-subject-out validation. In addition, the utilization of target-domain information varied across studies, including unsupervised domain adaptation, semi-supervised transfer, and supervised transfer settings. Some methods further incorporated calibration data to improve model adaptation. Meanwhile, studies also differed in baseline model selection, hyperparameter tuning, and uncertainty reporting. These experimental factors may affect the reliability of performance evaluation and the fairness of cross-study comparisons. Therefore, the results in Tables 4, 5 should be interpreted within their specific experimental protocols rather than being directly used for performance ranking or comparison across studies.

**Table 4:** Cross-subject results on BCI IV 2a.

Year	Paper	Model	Preprocessing	Train/Test splits	Accuracy	Target-label availability	Calibration-data usage	Validation procedure	Baseline configuration	Hyperparameter selection	Uncertainty statistics
2020	Zhang and Wu (2020)	MEKT	Band-Pass Filtering: 8–30 Hz	STS	68.73%	Unlabeled	NR	Fixed split	CSP-LDA, RA-MDM, RA-MDMCA, CA-CORAL, CA-GFK, CA-JDA, CA-JGSA	NR	NR
				MTS	76.31%						
2021	Zhu et al. (2021)	MFAR	Band-Pass Filtering: 8–30 Hz	MTS	72.15%	Unlabeled	√	NR	TCA, W-BDA, RA-MDRM, EA-CSP-wAR	NR	NR
2022	Gaur et al. (2022)	LR-TSTL	Band-Pass Filtering: 8–30 Hz	MTS	78.95%	Unlabeled	**×**	LOSO	M1, M2, M3, EEG-NAC, EEG-CSAC, EEGNet	NR	11.68%
	Chen et al. (2022a)	JDAOT-Mix	Band-Pass Filtering: 4–38 Hz	MTS	60.69%	Unlabeled	NR	LOSO	EA, TCA, JDA, BOTDA, DANN, CDAN, DJDAN	NR	NR
	Feng et al. (2022)	KMM-TrAda Boost	Band-Pass Filtering: 8–30 Hz	MTS	89.10%	Limited labeled	NR	LOSO	CSP + KMM, CSP + TrAdaBoost, CSP + SVM, et al.	NR	3.1%
	Zhang et al. (2022)	MSDT	Band-Pass Filtering: 8-32 Hz	MTS	76.54%	Unlabeled	NR	LOSO	CSP-LDA, DE-LR, EA-CSP-LDA, EEGNet, ShallowCNNSCM, DNN, DAN, DANN, CDAN, et al.	NR	NR
2023	Han et al. (2023)	GNNs and TL	Band-Pass Filtering: 4–40 Hz downsampled to 125 Hz Z-score normalization	MTS	72.50%	Limited labeled	NR	LOSO	ShallowCNN, DeepCNN, EEGNet, SBGCN	NR	9.63%
	She et al. (2023)	W-DAN	Band-Pass Filtering: 8-32 Hz	MTS	77.60%	Unlabeled	NR	LOSO	FBCSP, TLCsD, RA-MDRM, WTLT, EA-CSP-LDA, ConvNet, DRDA, DAFS	NR	10.85%
	Song et al. (2023)	GAT	Band-Pass Filtering:4–40 Hz Z-score normalization	MTS	76.58%	Limited labeled	NR	LOSO	FBCSP,ssCSP, SSMM, ConvNet, MI-CNN, C2CM, SHNN, DRDA	NR	NR
	Yin et al. (2024)	GITGAN	Band-Pass Filtering: 0.5-38 Hz	MTS	69.20%	Unlabeled	NR	LOSO	EIGITNet, EEGResNet, EEG-Adapt, ADAST, SLARDA, DeepConvNet	NR	12.99%
2024	Liang et al. (2024)	EISATC-Fusion	Z-score normalization	MTS	67.42%	Fully labeled	NR	LOSO	EEGNet-8,2, EEG-TCNet, MSFBCNN, LSTM-MLP-T, et al.	NR	10.85%
	Xu et al. (2024a)	FRCN	Band-Pass Filtering: 8–30 Hz Exponential Moving Standardization, EMS	MTS	63.70%	Unlabeled	NR	LOSO	FBCSP, MDM, ConvNet, C2CM, EEGNet, DRDA, DAFS, CBAM, GAT, CRAM, et al.	NR	NR
	Xu et al. (2024b)	RLPTL	Band-Pass Filtering: 8–30 Hz	STS	70.56%	Unlabeled	NR	Fixed split	RA-MDM, EA-CSP-LDA, RA-TCA, RA-EasyTL, RA-CORAL, RA-JDA, et al.	√	14.82%
				MTS	78.63%						11.79%
	Wang et al. (2024a)	ISMDA	NR	MTS	69.51%	Unlabeled	NR	LOSO	ShallowConvNet, EEGNet, EA-CSP, DRDA, MS-MDA, DAAN, CDAN, MCC	NR	NR
	Li et al. (2024b)	SAN	Band-Pass Filtering: 8–30 Hz	STS	77.15%	Unlabeled	NR	Fixed split	EEGNet, ConvNet, FBCSP, MSNN, Conformer, DANN, GConvNet	NR	NR
	Li et al. (2024c)	RCA-SPT	Band-Pass Filtering: 8–30 Hz SCM Vectorized SCM	MTS	76.02%	Limited labeled	NR	LOSO	TPT, PT-TPT, RCA-TPT, CSP, EA-CSP, Softmax, RCA-Softmax, SPT, PT-SPT	NR	NR
2025	Zhi et al. (2025)	SCLDGN	Euclidean-Space Alignment Band-Pass Filtering: 4–40 Hz	MTS	69.85%	Unlabeled	**×**	LOSO	EEGNet, Mixup, MMD, IRM, MLDG, DANN-MI, SelfReg	√	9.16%
	Jin et al. (2025)	MSTFNet	Band-Pass Filtering: 0.5–40 Hz	MTS	66.31%	Fully labeled	NR	LOSO	FBCSP, EEGNet, EEG-TCNet, FBCNet, ATCNet, D-ATCNet, DRDA,et al.	NR	NR
	Xu et al. (2025)	CLUDA	Band-Pass Filtering: 4–40 Hz Z-score standardization	MTS	58.37%	Unlabeled	NR	LOSO	EEGNet, ConvNet, Conformer, DRDA, DJDAN, Selective-MDA, DECISION, et al.	NR	NR
	Li et al. (2025)	MIFM	NR	MTS	74.55%	NR	NR	LOSO	Shallow ConvNet, Deep ConvNet, MAtt, ATCNet, MBK-CNN	NR	NR

^1^ STS, single-source to single-target; MTS, multi-source to single-target.

^2^ EMS, exponential moving standardization; SCM, sample covariance matrix; NR, not reported.

^3^ LOSO, leave-one-subject -out validation.

**Table 5:** Cross-session results on BCI IV 2a.

Year	Paper	Model	Preprocessing	Train/test splits	Accuracy	Target-label availability	Calibration-data usage	Validation procedure	Baseline configuration	Hyperparameter selection	Uncertainty statistics
2021	Zhao et al. (2021)	DRDA	Band-Pass Filtering: 8–30 Hz EMS	ses1 → ses2	74.75%	Fully labeled	NR	Fixed split	FBCSP, SMM, SSMM, MI-CNN, ConvNet, CCSP, SSCSP, HSS-ELM, TLCSD, et al.	NR	NR
2022	Zhang et al. (2023d)	MI-CAT	Band-Pass Filtering: 4–38 Hz EMS	ses1 → ses2	76.81%	Fully labeled	NR	Fixed split	FBCSP, SSMM, ConvNet, EEGNet, SHNN, CCSP, SSCSP, DRDA, DJDAN	NR	NR
	Tang et al. (2023)	RMRA	Vectorized SCM Band-Pass Filtering: 8–30 Hz	ses1 → ses2	77.02%	Unlabeled	NR	Fixed split	CNN, TCA, TJM, JDA, CORAL, RGF, MT, PT	√	NR
2024	Liang et al. (2024)	EISATC-Fusion	Z-score normalization	ses1 → ses2	84.57%	Fully labeled	NR	5-fold cross-validation	EEGNet-8,2, EEG-TCNet, Conformer, TSFCNet, Multi-branch, 3D CNN, et al.	NR	7.48%
	Xu et al. (2024a)	FRCN	Band-Pass Filtering: 8–30 Hz EMS	ses1 → ses2	79.40%	Unlabeled	NR	Fixed split	FBCSP, MDM, ConvNet, C2CM, EEGNet, DRDA, DAFS, CBAM, EEGNet-MDTL, GAT, CRAM	NR	NR
	Tao et al. (2024)	ADFCNN	Band-Pass Filtering: 0-40 Hz EMS	ses1 → ses2	79.39%	Fully labeled	NR	5-fold cross-validation	EEGNet, Deep ConvNet, Shallow ConvNet, FBCNet, IFNet, MCNN, MSHCNN, MMCNN	√	NR
	Zhang et al. (2024c)	GPL-UDAN	Band-Pass Filtering: 0-40 Hz EMS	ses1 → ses2	86.03%	Unlabeled	NR	Fixed split	FBCSP, ConvNet, EEGNet, SHNN, CCSP, SSCSP, DRDA, MI-DABAN, MI-CAT, et al.	√	11.92%
		GPL-SDAN			84.22%	Limited labeled					11.76%
	Li et al. (2024b)	SAN	Band-Pass Filtering: 8–30 Hz	ses1 → ses2	77.15%	Unlabeled	NR	Fixed split	EEGNet, ConvNet, FBCSP, Conformer, DANN, SAN, GConvNet	NR	NR
	Liu et al. (2024)	RGDDA Net	NR	ses1 → ses2	86.25%	Unlabeled	NR	Fixed split	FBCSP, EEGNet, ConvNet, SSD-SE-CNN, MIEEGNet, TS-SEFFNet, SHNN, DJDAN	NR	NR
2025	Jin et al. (2025)	MSTFNet	Band-Pass Filtering: 0.5–40 Hz	ses1 → ses2	83.62%	Fully labeled	NR	Fixed split	FBCSP, EEGNet, EEG-TCNet, FBCNet, ATCNet, D-ATCNet, Conformer, IDAN	NR	9.90%

^1^ EMS, exponential moving standardization; SCM, sample covariance matrix; NR, not reported.

^2^ ses1 → ses2, train with the first three sessions and test with the last two sessions.

#### 5.1.4 Methodological and reporting quality assessment

To evaluate the methodological reliability and evidence quality of the included studies, we established a methodological and reporting quality assessment framework based on the reported information in the original studies. Considering the variations in experimental settings and reporting practices across studies, the assessment was performed only based on explicitly reported information in the papers. Missing information was not inferred or estimated. The detailed assessment results are presented in Supplementary Table S5. We evaluated the quality of the included studies based on 11 dimensions, including data openness, code availability, statistical testing, cross-dataset validation, negative-transfer analysis, data leakage risk, split-protocol integrity, preprocessing transparency, fair baseline implementation, hyperparameter tuning, and calibration-data usage. Each dimension was scored according to the availability and completeness of the relevant information reported in the original studies. The operational criteria for each assessment dimension are provided in Supplementary Table S4.

For the Data Openness dimension, a score of 1 point was assigned when studies used publicly available datasets or provided sufficient descriptions of data collection procedures, experimental protocols, and data sources for in-house datasets. A score of 0 points was assigned when such information was insufficiently reported. For the other evaluation dimensions, a score of 1 point was assigned when the relevant experimental designs or analyses were explicitly reported in the original paper, marked as √. If the analysis was explicitly not conducted or the relevant information was not reported, the items were marked as × or Not Reported (NR) and assigned 0 points. If an evaluation dimension was not relevant to a study’s methodological design, it was marked as “Not Applicable (N/A)” and was not included in the total score calculation. For each study, the methodological and reporting quality score was calculated based on the applicable evaluation dimensions only, thereby avoiding inappropriate deductions caused by variations in study design. The calculation formula is defined as follows:


$$\text{Score}=\frac{\sum S_{i}}{N}\times 100\%$$

where $S_{i}$ represents the score of the i-th applicable evaluation dimension, and N represents the total number of applicable evaluation dimensions for the study.

Each evaluation dimension was independently assessed by two reviewers according to the predefined criteria. Any disagreements were resolved through discussion, with the involvement of a third reviewer when necessary. Based on the total scores, the included studies were categorized into three evidence quality levels: High (≥ 80%), Medium (>40 and <80%), and Low (≤ 40%). These thresholds were descriptively defined by the authors for the purpose of comparative evaluation. Higher scores indicate more complete methodological reporting, greater transparency of experimental procedures, and stronger evidence support. It should be noted that this grading system mainly reflects the quality of study reporting and the level of evidence support, rather than the performance superiority of the proposed methods.

Based on the predefined methodological and reporting quality assessment criteria, we evaluated the methodological quality of all 80 included studies. The results are presented in Supplementary Table S5. The quality assessment showed that 12 studies (15%) were classified as low evidence quality, 55 studies (68.75%) as medium evidence quality, and 13 studies (16.25%) as high evidence quality.

Overall, most studies demonstrated a moderate level of methodological reporting quality and provided basic information on experimental settings and evaluation procedures. However, several studies still had limitations in key factors that may affect the reliability of the reported results. Further analysis revealed that transparency of experimental procedures and reproducibility remain major limitations in current studies. Some studies did not sufficiently report hyperparameter selection strategies, calibration-data usage, or baseline model optimization procedures. Specifically, 66 studies did not clearly indicate whether hyperparameter tuning was performed using an independent validation set, 54 studies did not clearly describe the usage of target calibration data, and 31 studies did not report whether baseline methods were reasonably optimized. In addition, 13 studies did not explicitly state whether the train/test split followed subject-wise or independent-session protocols, indicating potential risks of data leakage.

Regarding experimental validation and data openness, only 8 studies used self-collected datasets, indicating that some researchers attempted to validate model performance under specific experimental scenarios. However, variations in data acquisition conditions and experimental protocols may increase the difficulty of comparing results across studies. In addition, 68 studies evaluated their methods on two or more datasets. This indicates that some included studies emphasized model generalization ability and assessed method stability through multi-dataset experiments. Regarding code availability, 26 studies provided their source codes, and some included studies emphasized research reproducibility through code sharing. However, the overall level of code sharing remains limited. A total of 47 studies performed statistical significance tests to support reliable performance comparisons. However, many studies still rely on evaluations using a single dataset. A total of 36 studies reported negative-transfer analysis, considering the effects of source-domain selection and domain differences on transfer performance and potential risks in transfer learning.

Overall, the included studies showed variability in experimental validation and result analysis procedures, and some methodological details remained insufficiently reported. Future cross-variability MI EEG research should further strengthen the standardization of validation procedures, the rigor of statistical analyses, the systematic evaluation of negative transfer, and the open sharing of data and codes. These improvements may enhance the reliability and generalizability of research findings. In the subsequent analyses, we also considered methodological quality differences among studies. Studies with higher evidence quality provide more reliable support, whereas findings from studies with lower evidence quality or insufficient reporting should be interpreted with caution.

### 5.2 Open problems and future trends

#### 5.2.1 Data openness

The limited availability of large-scale EEG datasets remains a major challenge for MI EEG cross-variability research. Existing studies mainly rely on public datasets such as BCI IV 2a and BCI IV 2b, which contain limited subjects and sessions. Although these datasets provide standardized evaluation platforms, their limited diversity may restrict the assessment of model generalization under real-world conditions.

To address data scarcity, large-scale and multi-session EEG data-sharing initiatives are gradually emerging. For example, the SHU dataset provides a more diverse benchmark by increasing the number of subjects and sessions, which supports the evaluation of cross-session and cross-subject generalization (Ma et al., 2022). Its further expansion increased the dataset scale and diversity, providing more comprehensive resources for MI EEG variability analysis (Yang et al., 2025b). In addition, data augmentation strategies provide another potential solution. Wang et al. (2024b) proposed a parameter-free Channel Reflection method that improves EEG data diversity by utilizing prior knowledge of channel distributions. Future studies should further establish larger, more diverse, and standardized EEG datasets while exploring effective augmentation strategies to enhance model robustness.

#### 5.2.2 User-friendliness

The practical application of MI EEG systems is limited by the difficulty of acquiring stable and high-quality EEG signals. Environmental noise, electrode impedance variations, and subject-dependent physiological changes can reduce signal reliability. Moreover, conventional wet-electrode systems require complex preparation procedures, which restrict portability and long-term usability.

To improve the accessibility and reliability of MI EEG acquisition, researchers have explored various hardware and acquisition technologies. Dry electrode technologies, including microneedle arrays, metallic contacts, and flexible substrates, can eliminate the need for conductive gels and improve user comfort and portability (Xing et al., 2018). Meanwhile, simplified acquisition schemes, such as single-electrode and reduced-channel EEG systems, have been investigated to reduce device complexity while maintaining acceptable signal quality (Gong et al., 2024). Future studies should focus on developing portable and user-friendly acquisition systems that balance signal quality, comfort, and practical usability.

#### 5.2.3 Privacy protection

EEG signals contain sensitive information related to individual identity, health conditions, and cognitive states (Zhang et al., 2022). In cross-variability research, the increasing use of multi-source EEG data introduces additional privacy risks during data sharing and utilization. Therefore, achieving effective knowledge transfer while protecting individual privacy remains an important challenge for practical MI EEG applications.

Privacy-preserving learning strategies provide potential solutions for balancing data utilization and privacy protection. Federated learning enables collaborative model training without exchanging raw EEG data, thereby reducing privacy risks while maintaining model performance (Liu and Wan, 2025). In addition, adversarial perturbation methods can protect sensitive information by modifying EEG representations while preserving task-related features (Aissa et al., 2025). Future studies should further investigate secure and efficient privacy-preserving frameworks for large-scale MI EEG data sharing and cross-variability learning.

#### 5.2.4 EEG foundation models for improving generalization capability

Most existing EEG decoding models are developed under specific datasets or experimental conditions, which limits their generalization across different subjects, sessions, and datasets. This lack of scalability remains a major challenge for developing universal EEG decoding frameworks.

Inspired by the success of large-scale pre-trained models after the release of ChatGPT in 2022 (OpenAI, 2023), EEG foundation models have emerged as a promising direction for improving EEG decoding generalization. By pre-training on large-scale and diverse EEG datasets, these models can learn more generalizable representations for various downstream tasks. Jiang et al. (2024) proposed LaBraM based on 16 public EEG datasets, demonstrating the potential of self-supervised pre-training for EEG representation learning. Brant (Zhang et al., 2023c) and Brant-X (Zhang et al., 2024b) further explored large-scale EEG and multi-physiological signal pre-training, providing new opportunities for developing more generalizable EEG decoding frameworks. Future studies should focus on constructing larger datasets and developing effective pre-training strategies to enhance the generalization capability of EEG models.

## 6 Conclusion

This review systematically summarizes the current progress of deep learning-based and transfer learning-based methods for cross-variability MI EEG decoding. It also analyzes the application characteristics of different approaches under various cross-variability scenarios. Based on a systematic analysis of 80 related studies, this review summarizes the existing advances from the perspectives of methodological mechanisms, applicable variability types, and experimental conditions. The reviewed studies indicate that deep learning-based methods provide richer feature learning strategies for MI EEG decoding by automatically learning spatiotemporal features, multi-scale information, and global context representations. Transfer learning-based methods show considerable potential for reducing discrepancies across different data distributions through mechanisms such as distribution alignment, feature transfer, and pseudo-label learning. However, performance comparisons among different methods should be interpreted with caution due to variations in dataset size, experimental protocols, target data usage, and evaluation criteria across existing studies. The quality assessment revealed that some included studies provided more comprehensive experimental transparency and generalization evaluation, although several studies still showed insufficient reporting regarding hyperparameter selection, calibration-data usage, fair baseline implementation, and data split protocols. Therefore, future studies should establish more standardized experimental procedures and evaluation criteria to improve the reliability and reproducibility of cross-variability MI EEG decoding research. Future efforts may focus on the construction of large-scale and diverse EEG datasets, more robust cross-domain adaptation strategies, privacy-preserving mechanisms, and EEG foundation models with stronger generalization capabilities.
