# Supplementary material for: Cross-variability decoding for motor imagery EEG signals: a comprehensive review
Source: Front Neurosci. 2026 Sep 8;20:1899645. doi: 10.3389/fnins.2026.1899645 (PMC13595434; doi:10.3389/fnins.2026.1899645)
Supplement: Supplementary file 1 [file Data_Sheet_1.PDF]

Cross-Variability Decoding for Motor Imagery EEG Signals: A Comprehensive Review

Lijun Wang [1], Yueying Zhou [1 *], Pengpai Wang [2], Xiujin Ma [1], Shufeng Zhou [1]

[1] School of Mathematics and Systems Science, Liaocheng University, Liaocheng 252000, China

[2] College of Computer and Information Engineering, Nanjing Tech University, Nanjing 211816, China

[*]Correspondence: zhouyueying@lcu.edu.cn

2 Materials and Methods

2.1 Literature Search

To improve the transparency and reproducibility of this systematic review, we provided detailed information on the search strategy and data extraction process in the supplementary materials. The search queries and field restrictions for each database are presented in Supplementary Table S1. The excluded studies during the full-text screening process and the corresponding reasons for exclusion are summarized in Supplementary Table S2. The extracted data from the included studies are provided in Supplementary Table S3.

TABLE S1
Search strategies and query terms used for literature retrieval in different databases

Databases	Field restrictions	Search strategy
IEEE Xplore	All Metadata	("All Metadata":"Cross Variability" OR "All Metadata":"Cross-Session" OR "All Metadata":"Subject-Independent" OR "All Metadata":"Cross-subject" OR "All Metadata":"Subject-Invariant") AND ("All Metadata":"domain adaptation" OR "All Metadata":"transfer learning" OR "All Metadata":"Deep Neural Network" OR "All Metadata":"Deep Learning" OR "All Metadata":"Deep Convolutional" OR "All Metadata":"Representation Learning") AND ("All Metadata":"EEG" OR "All Metadata":"Electroencephalography") AND ("All Metadata":"MI" OR "All Metadata":"Motor Imagery")
PubMed	Title/Abstract	("Cross Variability"[Title/Abstract] OR "Cross-Session"[Title/Abstract] OR "Subject-Independent"[Title/Abstract] OR "Cross-subject"[Title/Abstract] OR "Subject-Invariant"[Title/Abstract]) AND ("domain adaptation"[Title/Abstract] OR "transfer learning"[Title/Abstract] OR "Deep Neural Network"[Title/Abstract] OR "Deep Learning"[Title/Abstract] OR "Deep Convolutional"[Title/Abstract] OR "Representation Learning"[Title/Abstract]) AND ("EEG"[Title/Abstract] OR "Electroencephalography"[Title/Abstract]) AND ("MI"[Title/Abstract] OR "Motor Imagery"[Title/Abstract])

ScienceDirect	TITLE-ABS-KEY	TITLE-ABS-KEY("Cross Variability" OR "Cross-Session" OR "Subject-Independent" OR "Cross-subject" OR "Subject-Invariant") AND TITLE-ABS-KEY("domain adaptation" OR "transfer learning" OR "Deep Neural Network" OR "Deep Learning" OR "Deep Convolutional" OR "Representation Learning") AND TITLE-ABS-KEY("EEG" OR "Electroencephalography") AND TITLE-ABS-KEY("MI" OR "Motor Imagery")
Springer	Titles and keywords	("Cross Variability" OR "Cross-Session" OR "Subject-Independent" OR "Cross-subject" OR "Subject-Invariant") AND ("domain adaptation" OR "transfer learning" OR "Deep Neural Network" OR "Deep Learning" OR "Deep Convolutional" OR "Representation Learning") AND ("EEG" OR "Electroencephalography") AND ("MI" OR "Motor Imagery")

TABLE S2

Full-text exclusion table

Authors	Year	Title	Exclusion reason
M. Jiménez-Guarneros and P. Gómez-Gil	2020	Custom Domain Adaptation: A New Method for Cross-Subject, EEG-Based Cognitive Load Recognition	Not MI-EEG paradigm
J. M. Campbell, Z. Huang, J. Zhang, X. Wu, P. Qin, G. Northoff, G. A. Mashour and A. G. Hudetz	2020	Pharmacologically informed machine learning approach for identifying pathological states of unconsciousness via resting-state fMRI	Not MI-EEG paradigm
S.S. Mostafa, D. Baptista, A.G. Ravelo-García, G. Juliá-Serdá and F. Morgado-Dias	2020	Greedy based convolutional neural network optimization for detecting apnea	Not MI-EEG paradigm
M. Shen, B. Zou, X. Li, Y. Zheng, L. Li and L. Zhang	2021	Multi-source signal alignment and efficient multi-dimensional feature classification in the application of EEG-based subject-independent drowsiness detection	Not MI-EEG paradigm
J. L. Hagad, T. Kimura, K. I. Fukui and M. Numao	2021	Learning Subject-Generalized Topographical EEG Embeddings Using Deep Variational Autoencoders and Domain-Adversarial Regularization	Not MI-EEG paradigm
B. Almarri, S. Rajasekaran and C.H. Huang	2021	Automatic subject-specific spatiotemporal feature selection for subject-independent affective BCI	Not MI-EEG paradigm
W. Gao, T. Yu, J.G. Yu, Z. Gu, K. Li, Y. Huang and Y. Li	2021	Learning Invariant Patterns Based on a Convolutional Neural Network and Big Electroencephalography Data for Subject-Independent P300 Brain-Computer Interfaces	Not MI-EEG paradigm
J. Ma, Y. Lin, C. Hu, J. Zhang, Y. Yi and Z. Dai	2021	Integrated and segregated frequency architecture of the human brain network	Not MI-EEG paradigm
A. Zancanaro, G. Cisotto, J. R. Paulo, G. Pires and U. J. Nunes	2021	CNN-based Approaches For Cross-Subject Classification in Motor Imagery: From the State-of-The-Art to DynamicNet	Conference papers
C. Fan, J. Hu, S. Huang, Y. Peng and S. Kwong	2022	EEG-TNet: An End-To-End Brain Computer Interface Framework for Mental Workload Estimation	Not MI-EEG paradigm
R. Sharma, M. Kim and A. Gupta	2022	Motor imagery classification in brain-machine interface with machine learning algorithms: Classical approach to multi-layer perceptron model	Not new methods
F. Wu, W. Mai, Y. Tang, Q. Liu, J. Chen and Z. Guo	2022	Learning Spatial-Spectral-Temporal EEG Representations with Deep Attentive-Recurrent-Convolutional Neural Networks for Pain Intensity Assessment	Not MI-EEG paradigm
Y. Zhao, G. Dai, X. Fang, Z. Wu, N. Xia, Y. Jin and H. Zeng	2022	E3GCAPS: Efficient EEG-based multi-capsule framework with dynamic attention for cross-subject cognitive state detection	Not MI-EEG paradigm
J. Cui, Z. Lan, Y. Liu, R. Li, F. Li, O. Sourina and W. Müller-Wittig	2022	A compact and interpretable convolutional neural network for cross-subject driver drowsiness detection from single-channel EEG	Not MI-EEG paradigm
P. Peng, L. Xie, K. Zhang, J. Zhang, L. Yang and H. Wei	2022	Domain adaptation for epileptic EEG classification using adversarial learning and Riemannian manifold	Not MI-EEG paradigm
C.F. Vega, J. Quevedo, E. Escandón, M. Kiani, W. Ding and J. Andreu-Perez	2022	Fuzzy temporal convolutional neural networks in P300-based Brain–computer interface for smart home interaction	Not MI-EEG paradigm
Y. Zhao, G. Dai, X. Fang, Z. Wu, N. Xia, Y. Jin and H. Zeng	2022	E3GCAPS: Efficient EEG-based multi-capsule framework with dynamic attention for cross-subject cognitive state detection	Not MI-EEG paradigm
Z. Xu, Y. Zhou, X. Wen, Y. Niu, Z. Li, X. Xu, D. Zhang and X. Wu	2022	Cross subject personality assessment based on electroencephalogram functional connectivity and domain adaptation	Not MI-EEG paradigm

T. -J. Luo	2022	Dual regularized feature extraction and adaptation for cross-subject motor imagery EEG classification	Conference papers
T. Zaremba and A. Atyabi	2022	Cross-Subject & Cross-Dataset Subject Transfer in Motor Imagery BCI systems	Conference papers
T. D. Pham	2023	Classification of Motor-Imagery Tasks Using a Large EEG Dataset by Fusing Classifiers Learning on Wavelet-Scattering Features	Machine Learning
S. Zhang, Q. Wang, B. Zhang, Z. Liang, L. Zhang, L. Li, G. Huang, Z. Zhang, B. Feng and T. Yu	2023	Cauchy non-convex sparse feature selection method for the high-dimensional small-sample problem in motor imagery EEG decoding	Machine Learning
G. Huang, Z. Zhao, S. Zhang, Z. Hu, J. Fan, M. Fu, J. Chen, Y. Xiao, J. Wang and G. Dan	2023	Discrepancy between inter- and intra-subject variability in EEG-based motor imagery brain-computer interface: Evidence from multiple perspectives	Not new methods
E. M. Dos Santos, R. San-Martin and F. J. Fraga	2023	Comparison of subject-independent and subject-specific EEG-based BCI using LDA and SVM classifiers	Not new methods
I. Dolzhikova, B. Abibullaev and A. Zollanvari	2023	A Jackknife-Inspired Deep Learning Approach to Subject-Independent Classification of EEG	Not MI-EEG paradigm
R. Li, R. Gao, P. N. Suganthan, J. Cui, O. Sourina and L. Wang	2023	A spectral-ensemble deep random vector functional link network for passive brain–computer interface	Not MI-EEG paradigm
J. Choi, N. Kaongoen, H. Choi, M. Kim, B.H. Kim and S. Jo	2023	Decoding auditory-evoked response in affective states using wearable around-ear EEG system	Not MI-EEG paradigm
W. Huang, Z. Guan, K. Li, Y. Zhou and Y. Li	2023	An Affective Brain-Computer Interface Based on a Transfer Learning Method	Not MI-EEG paradigm
B. Li, J. Wang, Z. Guo and Y. Li	2023	Automatic detection of schizophrenia based on spatial–temporal feature mapping and LeViT with EEG signals	Not MI-EEG paradigm
S. Pain, S. Roy, M. Sarma and D. Samanta	2023	Detection of alcoholism by combining EEG local activations with brain connectivity features and Graph Neural Network	Not MI-EEG paradigm
N. Rahim, T. Abuhmed, S. Mirjalili, S. El-Sappagh and K. Muhammad	2023	Time-series visual explainability for Alzheimer‘s disease progression detection for smart healthcare	Not MI-EEG paradigm
X. Wu, Y. Feng, S. Lou, H. Zheng, B. Hu, Z. Hong and J. Tan	2023	Improving NeuCube spiking neural network for EEG-based pattern recognition using transfer learning	Not MI-EEG paradigm
J. Yang, Z. Zhang, Z. Fu, B. Li, P. Xiong and X. Liu	2023	Cross-subject classification of depression by using multiparadigm EEG feature fusion	Not MI-EEG paradigm
V.C. Caruso, A.H. Wray, E. Lescht and S.-E. Chang	2023	Neural oscillatory activity and connectivity in children who stutter during a non-speech motor task	Not MI-EEG paradigm
T. Duan, Z. Wang, S. Liu, Y. Yin and S.N. Srihari	2023	UNCER: A framework for uncertainty estimation and reduction in neural decoding of EEG signals	Not MI-EEG paradigm
A. Nagarajan, N. Robinson and C. Guan	2023	Relevance-based channel selection in motor imagery brain-computer interface	Not MI-EEG paradigm
T. -J. Luo	2023	An efficient domain adaptation framework for cross-subject EEG classification	Conference papers
Y. Wang, J. Wang, W. Wang, J. Su and Z. -G. Hou	2023	Calibration-Free Transfer Learning for EEG-Based Cross-Subject Motor Imagery Classification	Conference papers
M. Eder, J. Xu and M. Grosse-Wentrup	2024	Benchmarking brain-computer interface algorithms: Riemannian approaches vs convolutional neural networks	Not new methods
H. Kim, J. Kim and S. B. Kim	2024	Cross-subject generalizable representation learning with class-subject dual labels for biosignals	Not MI-EEG paradigm
S. Eldawlatly	2024	On the role of generative artificial intelligence in the development of brain-computer interfaces	Not new methods
Z. Guo, J. Wang, T. Jing and L. Fu	2024	Investigating the interpretability of schizophrenia EEG mechanism through a 3DCNN-based hidden layer features aggregation framework	Not MI-EEG paradigm
H. Li, G. Xu, C. Du, Z. Li, C. Han, P. Tian and S. Zhang	2024	Facilitating applications of SSVEP-BCI by effective Cross-Subject knowledge transfer	Not MI-EEG paradigm
X. Wang, M. Dang, K. Yang, X. Cui, D. Zhang and C. Chen	2024	The ensemble multi-scale convolution neural network for visual target detection EEG-based brain-computer interfaces	Not MI-EEG paradigm
A. Zaman, S. Kumar, S. Shatabda, I. Dehzangi and A. Sharma	2024	SleepBoost: A multi-level tree-based ensemble model for automatic sleep stage classification	Not MI-EEG paradigm
H. Sun, C. Li and H. Zhang	2024	Image Segmentation-P300 Selector: A Brain–Computer Interface System for Target Selection	Not MI-EEG paradigm
L. Yuan, J. Cui, R. Li, Z. Zheng, M.Y. Siyal and Z. Yi	2024	Entropy-guided robust feature domain adaptation for electroencephalogram-based cross-dataset drowsiness recognition	Not MI-EEG paradigm

C. Bunterngchit, T. Chearanai and Y. Bunterngchit	2024	Towards Robust Cross-Subject EEG-fNIRS Classification: A Hybrid Deep Learning Model with Optimized Feature Selection	Conference papers
Y. Hong, X. Zeng, F. Wu and J. Wang	2024	MAFNet: Multi-domain Features Attention-Based Fusion Network for Cross-Subject Motor Imagery Classification	Conference papers
S. An, M. Kang, S. Kim, P. Chikontwe, L. Shen and S.H. Park	2024	Subject-Adaptive Transfer Learning Using Resting State EEG Signals for Cross-Subject EEG Motor Imagery Classification	Conference papers
Y. Zhao, B. Jiang, Y. Yan, D. He, X. Zhang, S. Zou, Y. Zhu, X. Hu and G. Xie	2025	RGFTSLANet: A Cross-Subject Classification Model for Decoding EEG-Based Motor Imagery Tasks	Conference papers

TABLE S3
Supplementary extraction table covering the included studies

Authors	Year	Title	Cross-variability
L. Xu, M. Xu, Y. Ke, X. An, S. Liu and D. Ming	2020	Cross-Dataset Variability Problem in EEG Decoding With Deep Learning	cross-subject cross-dataset
X. Zhao, J. Zhao, C. Liu and W. Cai	2020	Deep Neural Network with Joint Distribution Matching for Cross-Subject Motor Imagery Brain-Computer Interfaces	cross-subject
K. Zhang, G. Xu, L. Chen, P. Tian, C. Han, S. Zhang and N. Duan	2020	Instance Transfer Subject-Dependent Strategy for Motor Imagery Signal Classification Using Deep Convolutional Neural Networks	cross-subject
W. Zhang and D. Wu	2020	Manifold Embedded Knowledge Transfer for Brain-Computer Interfaces	cross-subject
K. Zhang, N. Robinson, S.-W. Lee and C. Guan	2021	Adaptive transfer learning for EEG motor imagery classification with deep Convolutional Neural Network	cross-subject cross-session
H. Zhao, Q. Zheng, K. Ma, H. Li and Y. Zheng	2021	Deep Representation-Based Domain Adaptation for Nonstationary EEG Classification	cross-session
B. Sun, X. Zhao, H. Zhang, R. Bai and T. Li	2021	EEG Motor Imagery Classification With Sparse Spectrotemporal Decomposition and Deep Learning	cross-session
X. Liu, L. Lv, Y. Shen, P. Xiong, J. Yang and J. Liu	2021	Multiscale space-time-frequency feature-guided multitask learning CNN for motor imagery EEG classification	cross-subject
L. Zhu, J. Yang, W. Ding, J. Zhu, P. Xu, N. Ying and J. Zhang	2021	Multi-Source Fusion Domain Adaptation Using Resting-State Knowledge for Motor Imagery Classification Tasks	cross-subject
Y. Xu, X. Huang and Q. Lan	2021	Selective Cross-Subject Transfer Learning Based on Riemannian Tangent Space for Motor Imagery Brain-Computer Interface	cross-subject
X. Zhang, Q. She, Y. Chen, W. Kong and C. Mei	2021	Sub-band target alignment common spatial pattern in brain-computer interface	cross-subject
A. M. Roy	2022	Adaptive transfer learning-based multiscale feature fused deep convolutional neural network for EEG MI multiclassification in brain–computer interface	cross-subject cross-session
J. Feng, Y. Li, C. Jiang, Y. Liu, M. Li and Q. Hu	2022	Classification of motor imagery electroencephalogram signals by using adaptive cross-subject transfer learning	cross-subject
W. Zhang and D. Wu	2022	Lightweight Source-Free Transfer for Privacy-Preserving Motor Imagery Classification	cross-subject
P. Gaur, A. Chowdhury, K. McCreadie, R. B. Pachori and H. Wang	2022	Logistic Regression With Tangent Space-Based Cross-Subject Learning for Enhancing Motor Imagery Classification	cross-subject
P. Autthasan, R. Chaisaen, T. Sudhawiyangkul, P. Rangpong, S. Kiatthaveephong, N. Dilokthanakul, G. Bhakdisongkhram, H. Phan, C. Guan and T. Wilaiprasitporn	2022	MIN2Net: End-to-End Multi-Task Learning for Subject-Independent Motor Imagery EEG Classification	cross-subject
W. Zhang, Z. Wang and D. Wu	2022	Multi-Source Decentralized Transfer for Privacy-Preserving BCIs	cross-subject
K. Xia, L. Deng, W. Duch and D. Wu	2022	Privacy-Preserving Domain Adaptation for Motor Imagery-Based Brain-Computer Interfaces	cross-subject cross-session
Z. Wan, R. Yang, M. Huang, F. E. Alsaadi, M. M. Sheikh and Z. Wang	2022	Segment alignment based cross-subject motor imagery classification under fading data	cross-subject

Y. Chen, R. Yang, M. Huang, Z. Wang and X. Liu	2022	Single-Source to Single-Target Cross-Subject Motor Imagery Classification Based on Multisubdomain Adaptation Network	cross-subject
V. Peterson, N. Nieto, D. Wyser, O. Lambercy, R. Gassert, D. H. Milone and R. D. Spies	2022	Transfer Learning Based on Optimal Transport for Motor Imagery Brain-Computer Interfaces	cross-session
P. Chen, H. Wang, X. Sun, H. Li, C. Grebogi and Z. Gao	2022	Transfer Learning With Optimal Transportation and Frequency Mixup for EEG-Based Motor Imagery Recognition	cross-subject
X. C. Zhong, Q. Wang, D. Liu, J. X. Liao, R. Yang, S. Duan, G. Ding and J. Sun	2023	A deep domain adaptation framework with correlation alignment for EEG-based motor imagery classification	cross-subject<br>cross-session
H. Jia, S. Yu, S. Yin, L. Liu, C. Yi, K. Xue, F. Li, D. Yao, P. Xu and T. Zhang	2023	A Model Combining Multi Branch Spectral-Temporal CNN, Efficient Channel Attention, and LightGBM for MI-BCI Classification	cross-session
J. Luo, Y. Wang, S. Xia, N. Lu, X. Ren, Z. Shi and X. Hei	2023	A shallow mirror transformer for subject-independent motor imagery BCI	cross-subject
N. Mammone, C. Ieracitano, H. Adeli and F. C. Morabito	2023	AutoEncoder Filter Bank Common Spatial Patterns to Decode Motor Imagery From EEG	cross-subject
T. J. Luo	2023	Dual selections based knowledge transfer learning for cross-subject motor imagery EEG classification	cross-subject
H. Altaheri, G. Muhammad and M. Alsulaiman	2023	Dynamic Convolution With Multilevel Attention for EEG-Based Motor Imagery Decoding	cross-subject
J. Han, X. Wei and A. A. Faisal	2023	EEG decoding for datasets with heterogenous electrode configurations using transfer learning graph neural networks	cross-subject<br>cross-dataset
R. R. Chowdhury, Y. Muhammad and U. Adeel	2023	Enhancing Cross-Subject Motor Imagery Classification in EEG-Based Brain-Computer Interfaces by Using Multi-Branch CNN	cross-subject
M. Miao, Z. Yang, H. Zeng, W. Zhang, B. Xu and W. Hu	2023	Explainable cross-task adaptive transfer learning for motor imagery EEG classification	cross-task<br>cross-subject
K. Yin, E. Y. Lim and S.-W. Lee	2023	GITGAN: Generative inter-subject transfer for EEG motor imagery analysis	cross-subject
Y. Song, Q. Zheng, Q. Wang, X. Gao and P. A. Heng	2023	Global Adaptive Transformer for Cross-Subject Enhanced EEG Classification	cross-subject
Y. Zhang, Y. Yu, B. Wang, H. Shen, G. Lu, Y. Liu, L. L. Zeng and D. Hu	2023	Graph Learning With Co-Teaching for EEG-Based Motor Imagery Recognition	cross-subject
P. Deny, S. Cheon, H. Son and K. W. Choi	2023	Hierarchical Transformer for Motor Imagery-Based Brain Computer Interface	cross-subject
Q. She, T. Chen, F. Fang, J. Zhang, Y. Gao and Y. Zhang	2023	Improved Domain Adaptation Network Based on Wasserstein Distance for Motor Imagery EEG Classification	cross-subject<br>cross-session
A. Li, Z. Wang, X. Zhao, T. Xu, T. Zhou and H. Hu	2023	MDTL: A Novel and Model-Agnostic Transfer Learning Strategy for Cross-Subject Motor Imagery BCI	Cross-Subject
D. Zhang, H. Li and J. Xie	2023	MI-CAT: A transformer-based domain adaptation network for motor imagery classification	cross-session
E. Jeon, W. Ko, J. S. Yoon and H. I. Suk	2023	Mutual Information-Driven Subject-Invariant and Class-Relevant Deep Representation Learning in BCI	cross-subject<br>cross-session
H. Altaheri, G. Muhammad and M. Alsulaiman	2023	Physics-Informed Attention Temporal Convolutional Network for EEG-Based Motor Imagery Classification	cross-subject
X. Zhang, Z. Miao, C. Menon, Y. Zheng, M. Zhao and D. Ming	2023	Priming cross-session motor imagery classification with a universal deep domain adaptation framework	cross-session
L. Pan, K. Wang, L. Xu, X. Sun, W. Yi, M. Xu and D. Ming	2023	Riemannian geometric and ensemble learning for decoding cross-session motor imagery electroencephalography signals	cross-session
P. Saideepthi, A. Chowdhury, P. Gaur and R. B. Pachori	2023	Sliding Window Along With EEGNet-Based Prediction of EEG Motor Imagery	cross-subject<br>cross-session
Y. Dong, X. Wen, F. Gao, C. Gao, R. Cao, J. Xiang and R. Cao	2023	Subject-Independent EEG Classification of Motor Imagery Based on Dual-Branch Feature Fusion	cross-subject
J. Bi and M. Chu	2023	TDLNet: Transfer Data Learning Network for Cross-Subject Classification Based on Multiclass Upper Limb Motor Imagery EEG	cross-subject

X. Tang, X. Li, W. Li, B. Hao, Y. Xie and X. Dang	2023	Transfer Learning: Rotation Alignment With Riemannian Mean for Brain–Computer Interfaces and Wheelchair Control	cross-subject cross-session
W. Liu, C. Guo and C. Gao	2024	A cross-session motor imagery classification method based on Riemannian geometry and deep domain adaptation	cross-session
J. Xu, D. Li, P. Zhou, Y. Zhang, Z. Wang and D. Ma	2024	A Relation Feature Comparison Network for Cross-Domain Recognition of Motion Intention	cross-subject cross-session
W. Tao, Z. Wang, C. M. Wong, Z. Jia, C. Li, X. Chen, C. L. P. Chen and F. Wan	2024	ADFCNN: Attention-Based Dual-Scale Fusion Convolutional Neural Network for Motor Imagery Brain–Computer Interface	cross-session
X. Chen, Z. Wang and D. Wu	2024	Alignment-Based Adversarial Training (ABAT) for Improving the Robustness and Accuracy of EEG-Based BCIs	cross-session
S. An, S. Kim, P. Chikontwe and S. H. Park	2024	Dual Attention Relation Network With Fine-Tuning for Few-Shot EEG Motor Imagery Classification	cross-subject cross-dataset
J. Zhu, G. Xu, Q. Hu, B. Wang, T. Zhou and J. Qin	2024	Dual Contrastive Training and Transferability-Aware Adaptation for Multisource Privacy-Preserving Motor Imagery Classification	cross-subject
T.-j. Luo	2024	Dual regularized spatial-temporal features adaptation for multi-source selected cross-subject motor imagery EEG classification	cross-subject
C. Olvera, O. M. Ross and Y. Rubio	2024	EEG-based motor imagery classification with quantum algorithms	cross-subject
H. Wang, P. Chen, M. Zhang, J. Zhang, X. Sun, M. Li, X. Yang and Z. Gao	2024	EEG-Based Motor Imagery Recognition Framework via Multisubject Dynamic Transfer and Iterative Self-Training	cross-subject
G. Liang, D. Cao, J. Wang, Z. Zhang and Y. Wu	2024	EISATC-Fusion: Inception Self-Attention Temporal Convolutional Network Fusion for Motor Imagery EEG Decoding	cross-subject cross-session
H. Sun, Y. Ding, J. Bao, K. Qin, C. Tong, J. Jin and C. Guan	2024	Leveraging temporal dependency for cross-subject-MI BCIs by contrastive learning and self-attention	cross-subject
H. Wu, S. Li and D. Wu	2024	Motor Imagery Classification for Asynchronous EEG-Based Brain–Computer Interfaces	cross-subject cross-session
S. Yu, Z. Wang, F. Wang, K. Chen, D. Yao, P. Xu, Y. Zhang, H. Wang and T. Zhang	2024	Multiclass classification of motor imagery tasks based on multi-branch convolutional neural network and temporal convolutional network model	cross-session
M. Lee, H. Y. Park, W. Park, K. T. Kim, Y. H. Kim and J. H. Jeong	2024	Multi-Task Heterogeneous Ensemble Learning-Based Cross-Subject EEG Classification Under Stroke Patients	cross-subject
S. Asgarian, Z. Wang, F. Wan, C. M. Wong, F. Liu, Y. Mohsenzadeh, B. Wang and C. X. Ling	2024	Multiview Contrastive Learning for Unsupervised Domain Adaptation in Brain–Computer Interfaces	cross-session
J. Khabti, S. AlAhmadi and A. Soudani	2024	Optimal Channel Selection of Multiclass Motor Imagery Classification Based on Fusion Convolutional Neural Network with Attention Blocks	cross-subject cross-session
X. Li, X. Tang, J. Liu, Q. Liu, H. Wang and K. Liu	2024	Parameter transfer and Riemannian space coordinate alignment for EEG intention recognition	cross-subject
G. Xu, Z. Wang, H. Hu, X. Zhao, R. Li, T. Zhou and T. Xu	2024	Riemannian Locality Preserving Method for Transfer Learning With Applications on Brain-Computer Interface	cross-subject
F. Zhuo, X. Zhang, F. Tang, Y. Yu and L. Liu	2024	Riemannian transfer learning based on log-Euclidean metric for EEG classification	cross-subject cross-session
J. Lee, J. W. Choi and S. Jo	2024	Selective Multi-Source Domain Adaptation Network for Cross-Subject Motor Imagery Discrimination	cross-subject
W. Li, H. Li, X. Sun, H. Kang, S. An, G. Wang and Z. Gao	2024	Self-supervised contrastive learning for EEG-based cross-subject motor imagery recognition	cross-subject
X. Li, X. Tang, S. Qiu, X. Deng, H. Wang and Y. Tian	2024	Subdomain Adversarial Network for Motor Imagery EEG Classification Using Graph Data	cross-subject cross-session
S. Sartipi and M. Cetin	2024	Subject-Independent Deep Architecture for EEG-Based Motor Imagery Classification	cross-subject
H. W. Ng and C. Guan	2024	Subject-independent meta-learning framework towards optimal training of EEG-based classifiers	cross-subject
J. Zhang, J. Fang, S. Liu, D. Liu, H. Wu and J. Long	2024	Towards Cross-Brain Computer Interface: A Prototype-Supervised Adversarial Transfer Learning Approach with Multiple Sources	cross-subject

A. Nagarajan, N. Robinson, K. K. Ang, K. S. G. Chua, E. Chew and C. Guan	2024	Transferring a deep learning model from healthy subjects to stroke patients in a motor imagery brain-computer interface	cross-subject
D. Zhang, H. Li and J. Xie	2024	Unsupervised and semi-supervised domain adaptation networks considering both global knowledge and prototype-based local class information for Motor Imagery Classification	cross-session
Z. Wang, H. Wang, T. Jia, X. He, S. Li, D. Wu	2025	DBConformer: Dual-Branch Convolutional Transformer for EEG Decoding	cross-subject<br>cross-session
Wang, J.; Zhao, S.; Luo, Z.; Zhou, Y.; Li, S.; Pan, G.	2025	EEGMamba: An EEG Foundation Model with Mamba.	cross-subject<br>cross-dataset
S. Yang, Z. Huang and T.-J. Luo	2025	Joint spatial feature adaption and confident pseudo-label selection for cross-subject motor imagery EEG signals classification	cross-subject
H. Li, J. An, R. Juan, T.P. Benko, M. Perc, W. Dang and Z. Gao	2025	Manifold-based multi-branch transfer learning for MI-EEG decoding	cross-subject
J. Jin, W. Chen, R. Xu, W. Liang, X. Wu, X. He, X. Wang, A. Cichocki.	2025	Multiscale spatial-temporal feature fusion neural network for motor imagery brain-computer interfaces	cross-subject<br>cross-session
H. Zhi, T. Yu, Z. Gu, Z. Lin, L. Che, Y. Li and Z. Yu	2025	Supervised Contrastive Learning-Based Domain Generalization Network for Cross-Subject Motor Decoding	cross-subject
C. Xu, Y. Song, Q. Zheng, Q. Wang and P.-A. Heng	2025	Unsupervised multi-source domain adaptation via contrastive learning for EEG classification	cross-subject

5 Discussion

5.1 Discussion

5) Methodological and reporting quality assessment

Table S4 summarizes the operational criteria used to determine the assessment outcomes (√, ×, NR, and N/A) for each of the 11 methodological and reporting quality dimensions. These criteria were predefined to ensure consistent and reproducible evaluation across the included studies. A criterion was marked as N/A when it was not applicable to the study design, while NR was assigned when the relevant information was not reported in the original manuscript.

TABLE S4
Operational criteria for determining assessment outcomes of the methodological and reporting quality evaluation

Assessment dimension	√ (Criterion satisfied)	× (Criterion not satisfied)	NR (Not reported)	N/A (Not applicable)
Data Openness	The dataset source was publicly available, or sufficient information about in-house dataset.	Only an in-house dataset was used, and insufficient information regarding the dataset source or collection procedure was provided.	Datasets used for validation was not clearly reported	No EEG dataset was used in the study.
Code Availability	Code or sufficient implementation details were provided.	Code was unavailable and implementation details were insufficient.	Code availability was not mentioned.	No independent computational method was developed or evaluated.
Statistical Testing	Statistical tests or uncertainty analysis were reported.	Statistical comparison was performed without appropriate statistical analysis.	Statistical analysis was not reported.	No quantitative comparison or statistical evaluation was performed.

Cross-Dataset Validation	The proposed method was evaluated using two or more independent datasets.	Only a single dataset was used for validation.	Datasets used for validation was not clearly reported.	No EEG dataset was used in the study.
Negative-Transfer Analysis	Negative transfer or transfer limitations were analyzed.	Transfer learning was applied without analyzing negative transfer.	Negative-transfer analysis was not reported.	No transfer learning or domain adaptation was involved.
Data Leakage Prevention	Data partitioning and experimental procedures were clearly described to reduce leakage risk.	Potential leakage existed or prevention strategies were insufficient.	Leakage-related information was not reported.	No machine learning-based evaluation was performed.
Split-protocol integrity	Training/testing procedures were clearly described and appropriate.	Data splitting strategy was unclear.	Data splitting protocol was not reported.	No model training/testing procedure was involved.
Preprocessing transparency	EEG preprocessing procedures were sufficiently described.	Important preprocessing information was incomplete.	Preprocessing procedures were not reported.	Preprocessing was outside the study scope.
Fair baseline implementation	Appropriate baseline methods were included for comparison.	Baseline comparison was insufficient or unfair.	Baseline information was not reported.	The study was not designed for method comparison.
Hyperparameter tuning	Hyperparameter selection or optimization strategy was reported.	Parameter tuning was performed without describing the strategy.	Hyperparameter selection was not reported.	Fixed parameters were used without optimization.
Calibration-data usage	Target-domain calibration data usage was explicitly described.	Calibration data were required but insufficiently described.	Calibration-data usage was not reported.	No target-domain calibration or adaptation was involved.
