# Supplementary material for: Cross-variability decoding for motor imagery EEG signals: a comprehensive review
Source: Front Neurosci. 2026 Sep 8;20:1899645. doi: 10.3389/fnins.2026.1899645 (PMC13595434; doi:10.3389/fnins.2026.1899645)
Supplement: Supplementary file 2 [file data_sheet_2.docx]

**TABLE S5**

Methodological and reporting quality assessment criteria of the 80 included studies

| **Year** | **Paper** | **Model** | **Data Openness** | **Data Openness** | **Statistical Testing** | **Cross-Dataset Validation** | **Negative-Transfer Analysis** | **Data Leakage Prevention** | **Split-protocol integrity** | **Preprocessing transparency** | **Fair baseline implementation** | **Hyperparameter tuning** | **Calibration-data usage** | **Evidence strength** |
| --- | --- | --- | --- | --- | --- | --- | --- | --- | --- | --- | --- | --- | --- | --- |
| 2020 | *Zhang & Wu, 2020* | MEKT | BCI IV 1  BCI IV 2a | https://github.com/chamwen/MEKT | Paired t-test | √ | √ | **√** | √ | √ | √ | NR | NR | High |
|  | *Zhang et al., 2020* | ITSD | BCI IV 2b | NR | ANOVA | × | √ | **√** | NR | √ | NR | NR | × | Low |
|  | *Zhao et al., 2020* | DCJNN | BCI IV 2a  GIST | NR | NR | √ | NR | **√** | √ | √ | √ | √ | NR | Medium |
|  | *Xu et al., 2020* | Online pre-alignment | BCI IV 2a  BCI IV 2b  PhysioNet  GIST  Weibo2014  Zhou2016  CBCIC2019001  CBCIC2019004 | NR | Wilcoxon signed-rank test | √ | N/A | **×** | √ | √ | √ | √ | N/A | High |
| 2021 | *Zhao et al., 2021* | DRDA | BCI IV 2a  BCI IV 2b | NR | NR | √ | √ | **√** | √ | √ | √ | NR | NR | Medium |
|  | *Zhu et al., 2021* | MFAR | BCI IV 1  BCI IV 2a | NR | Paired t-test | √ | √ | **√** | √ | √ | √ | NR | NR | Medium |
|  | *Zhang et al., 2021a* | SBTACSP | BCI IV 2a  BCI IV 2b | NR | NR | √ | NR | **×** | √ | √ | NR | N/A | N/A | Medium |
|  | Zhang et al.,2021b | NR | OpenBMI | https://github.com/zhangks98/eeg-adapt | Paired t-test | × | NR | **√** | √ | √ | √ | √ | × | Low |
|  | *Xu,Huang&Lan, 2021* | sSCSTL | GIST  BCI IV 1 | NR | Paired t-test | √ | √ | **×** | √ | √ | √ | NR | √ | High |
|  | *Sun et al., 2021* | SSD-SE | BCI IV 2b  in-house dataset | NR | T-test | √ | √ | **×** | √ | √ | √ | √ | × | Medium |
|  | *Liu et al., 2021* | MSML | BCI IV 2a  BCI IV 2b  HGD | https://github.com/lvlinyang/EEGclassification | NR | √ | N/A | **√** | √ | √ | NR | NR | NR | Medium |
| 2022 | *Wan et al., 2022* | SA-FBCSP-SVM | BCI IV 2a  in-house dataset | NR | NR | √ | N/A | **√** | √ | √ | √ | N/A | N/A | Medium |
| 2022 | *Gaur et al., 2022* | LR-TSTL | BCI IV 2a  Chowdhury et al. | NR | Wilcoxon signed-rank test | √ | √ | **√** | √ | √ | NR | NR | N/A | Medium |
|  | *Chen et al., 2022a* | JDAOT-Mix | BCI IV 2a  BCI IV 2b  CLA MI | NR | NR | √ | NR | **√** | √ | √ | √ | NR | NR | Medium |
|  | *Feng et al., 2022* | KMM-TrAdaBoost | BCI IV 2a  BCI IV 1  in-house dataset | NR | Paired t-test | √ | NR | **×** | √ | √ | √ | NR | NR | Medium |
|  | *Zhang, Wang & Wu , 2022* | MSDT | BCI IV 2a | NR | NR | √ | √ | **√** | √ | √ | √ | NR | NR | Medium |
|  | *Xia et al., 2022* | ASFA | BCI IV 2a  BNCI2014002  BNCI2015001 | NR | Paired t-test | √ | √ | **√** | √ | √ | √ | NR | NR | Medium |
|  | *Chen et al., 2022b* | MSDAN | BCI III 4a | NR | Paired t-test | NR | NR | **√** | √ | √ | √ | NR | NR | Medium |
|  | *Peterson et al., 2022* | BOTDA | Peterson et al.  BNCI2015001 | https://github.com/vpeterson/otda-mibci | non-parametric Friedman test  post-hoc Nemenyi test | √ | NR | **√** | √ | √ | √ | NR | NR | Medium |
|  | Roy, 2022 | MSFFCNN | BCI IV 2a | NR | NR | NR | NR | **√** | √ | √ | √ | NR | NR | Low |
|  | *Zhang & Wu, 2022* | LSFT | BCI IV 2a  BNCI2015001  AlexMI | NR | NR | √ | √ | **√** | √ | √ | √ | √ | NR | Medium |
|  | *Autthasan et al., 2022* | MIN2Net | BCI IV 2a  SMR-BCI  OpenBMI | https://github.com/IoBT-VISTEC/MIN2Net | Paired t-test | √ | √ | **√** | √ | √ | √ | √ | NR | Medium |
|  | *Zhang, Li & Xie, 2023* | MI-CAT | BCI IV 2a  BCI IV 2b | NR | NR | √ | N/A | **√** | √ | √ | √ | NR | N/A | High |
| 2023 | *Tang et al., 2023* | RMRA | BCI IV 2a  BCI IV 2b  in-house dataset | https://github.com/starchen-li/RotationAlignment-with-Riemannian-Mean-RMRA | ANOVA | √ | √ | **√** | √ | √ | √ | NR | NR | High |
|  | *Han, Wei & Faisal, 2023* | GNNs and TL | BCI IV 2a  PhysioNet  OpenBMI | NR | T-test | √ | √ | **√** | √ | √ | √ | NR | NR | Medium |
|  | *She et al., 2023* | W-DAN | BCI IV 2a  BCI IV 2b | NR | T-test | √ | √ | **√** | √ | √ | √ | NR | NR | Medium |
|  | *Song et al., 2023* | GAT | BCI IV 2a  BCI IV 2b | NR | Wilcoxon signed-rank test | √ | √ | **√** | √ | √ | √ | NR | NR | Medium |
|  | *Mammone et al., 2023* | AE-FBCSP | PhysioNet | NR | Wilcoxon rank-sum Test | × | √ | **√** | √ | √ | √ | √ | NR | High |
|  | *Pan et al., 2023* | RAVE | in-house dataset  BCI IV 2a  BNCI2015001  BNCI2015004 | https:// github.com/PLC-TJU/RAVE  https://github.com/ TBC-TJU/MetaBCI | T-test | √ | NR | **√** | √ | √ | √ | N/A | NR | Medium |
| 2023 | *Bi & Chu, 2023* | TDLNet | in-house dataset  GRAZ Dataset | NR | NR | √ | NR | **√** | √ | √ | √ | NR | N/A | High |
|  | *Li et al., 2023* | MDTL | BCI IV 2a  OpenBMI | NR | Wilcoxon signed-rank test | √ | NR | **×** | √ | √ | √ | NR | N/A | Medium |
|  | *Jeon et al., 2023* | SICR | GIST  KU | https://github.com/ku-milab/SICR_BCI | Wilcoxon signed-rank test | √ | √ | **×** | √ | √ | √ | √ | NR | Medium |
|  | *Zhong et al., 2023* | DDAF-CORAL | BCI II 3  BCI III 4a  BCI IV 2b | NR | NR | √ | √ | **×** | √ | √ | √ | NR | NR | High |
|  | *Zhang et al.,2023a* | SDDA | BCI IV 2a  BCI IV 2b | NR | NR | √ | NR | **√** | √ | √ | √ | √ | NR | Low |
|  | *Luo, 2023* | DS-KTL | BCI IV 2a  BCI IV 2b | NR | T-test | √ | √ | **√** | √ | √ | √ | NR | NR | Medium |
|  | *Miao et al., 2023* | ExCTATL | HGD  OpenBMI  GIST | https://github.com/bciResearcher/CT-adaptTL | Paired t-test | √ | √ | **√** | √ | √ | √ | √ | NR | Medium |
|  | *Zhang et al., 2023b* | CTGL | PhysioNet  HGD | NR | NR | √ | √ | **×** | √ | √ | √ | NR | NR | High |
|  | *Jia et al., 2023* | MBSTCNN-EL | BCI IV 2a | https://github.com/ microsoft/LightGBM | Paired t-test | × | √ | **√** | √ | √ | √ | NR | NR | Medium |
|  | *Chowdhury, Muhammad & Adeel, 2023* | EEGNet Fusion V2 | PhysioNet  BCI IV 2a  BCI IV 2b | https://github.com/radiarayan-chowdhury/EEGNet-Fusion-V2 | NR | √ | N/A | **×** | √ | √ | √ | NR | NR | Medium |
|  | *Deny et al., 2023* | HT | BCI IV 2a  PhysioNet  GIST  OpenBMI | NR | NR | √ | N/A | **×** | √ | √ | √ | NR | NR | Medium |
| 2023 | *Altaheri, Muhammad & Alsulaiman, 2023a* | ATCNet | BCI IV 2a | NR | NR | × | N/A | **√** | √ | √ | √ | N/A | N/A | Medium |
|  | *Altaheri, Muhammad & Alsulaiman, 2023b* | D-ATCNet | BCI IV 2a | https://github.com/altaheri | NR | × | N/A | **√** | √ | √ | √ | NR | N/A | Medium |
|  | *Saideepthi et al., 2023* | EEGNet + LCR | CBCIC2020  BCI IV 2b | NR | Wilcoxon signed-rank test | √ | N/A | **√** | √ | √ | √ | √ | N/A | Medium |
|  | *Luo et al., 2023* | SMT | BCI IV 2a  BCI IV 2b  OpenBMI | https://github.com/roniusLuo/shallowMirrorTransformer | NR | √ | N/A | **×** | √ | √ | √ | NR | N/A | High |
|  | *Dong et al., 2023* | MSAENet | BCI IV 2a  SMR-BCI  OpenBMI | NR | NR | √ | N/A | **√** | √ | √ | √ | NR | N/A | Medium |
|  | *Yin, Lim & Lee, 2023* | GITGAN | OpenBMI  BCI IV 2a | https://github.com/Kang1121/GITGAN | Wilcoxon signed-rank test | √ | N/A | **√** | √ | √ | √ | NR | N/A | Medium |
| 2024 | *Xu et al., 2024a* | FRCN | BCI IV 2a  BCI IV 2b | NR | NR | √ | √ | **√** | √ | √ | √ | NR | NR | Medium |
|  | *Liang et al., 2024* | EISATC-Fusion | BCI IV 2a  BCI IV 2b | https://github.com/LiangXiaohan506/ EISATC-Fusion | Wilcoxon signed-rank test | √ | √ | **√** | √ | √ | √ | √ | NR | High |
|  | *Xu et al., 2024b* | RLPTL | BCI IV 2a  BCI IV 1  GIST | NR | Paired t-test | √ | √ | **√** | √ | √ | √ | NR | NR | Medium |
|  | *Wang et al., 2024a* | ISMDA | BCI IV 2a  HGD  OpenBMI | NR | Wilcoxon signed-rank test | √ | √ | **√** | √ | √ | √ | NR | N/A | High |
| 2024 | *Li et al., 2024c* | RCA-SPT | BCI IV 2a  BCI IV 2b  BNCI2015001 | https://github.com/LaboratoryofCSBC/RCA-and-Parameter-Transfer. | Paired t-test | √ | √ | **√** | √ | √ | NR | NR | × | Medium |
|  | *Tao et al., 2024* | ADFCNN | BCI IV 2a  BCI IV 2b  OpenBMI | https://github.com/UM-Tao/ADFCNN-MI | Wilcoxon signed-rank test | √ | √ | **√** | √ | √ | √ | NR | × | Medium |
|  | *Zhang, Li & Xie, 2024* | GPL | BCI IV 2a  GIST | https://github.com/zhangdx21/GPL | NR | √ | NR | **√** | √ | √ | NR | NR | × | Medium |
|  | *Li et al., 2024b* | SAN | BCI IV 2a  BNCI2015001 | NR | NR | √ | √ | **√** | √ | √ | NR | NR | × | Medium |
|  | *Liu, Guo & Gao, 2024* | RGDDANet | BCI IV 2a  BCI IV 2b | NR | NR | √ | √ | **√** | √ | √ | NR | NR | × | Medium |
|  | *Lee et al., 2024* | MHEL | in-house dataset | × | Two-way ANOVA  One-way ANOVA  Paired t-test | × | NR | **√** | √ | √ | NR | NR | × | Low |
|  | *Chen, Wang & Wu, 2024* | ABAT | BCI IV 2a  MI6  MI2 | https://github.com/ xqchen914/ABAT | NR | √ | × | **√** | √ | √ | NR | NR | N/A | Low |
|  | *Lee, Choi & Jo., 2024* | Selective-MDA | BCI IV 2a  ARA | NR | NR | √ | NR | **√** | √ | √ | NR | NR | × | Medium |
|  | *Zhang et al., 2024a* | PSAT | BCI IV 2a  BCI IV 2b  BCI IV 1 | https://jiazhang-ml.pub/PSAT-main.zip | Paired t-test | √ | √ | **√** | √ | √ | NR | NR | × | Medium |
|  | *Sartipi & Cetin., 2024* | SSDA | PhysioNet  BCI IV 2a | NR | Wilcoxon signed-rank test | √ | √ | **√** | √ | √ | NR | NR | NR | Medium |
|  | *Luo, 2024* | DRSTFA | BCI IV 2a  BCI IV 2b  BCI III 4a | NR | Paired t-test | √ | NR | **√** | √ | √ | NR | NR | N/A | Medium |
|  | *An et al., 2024* | DA-RelationNet | BCI IV 2a  BCI IV 2b  GIST | NR | NR | √ | √ | **√** | √ | √ | NR | NR | NR | Medium |
| 2024 | *Asgarian et al., 2024* | MACNet | BCI IV 2a  OpenBMI  SMR | NR | NR | √ | NR | **√** | √ | NR | NR | NR | NR | Low |
|  | *Zhuo et al., 2024* | LEM | BCI III 3a  BCI IV 2a | NR | NR | √ | NR | **√** | √ | √ | NR | √ | NR | Low |
|  | *Ng & Guan., 2024* | NR | BCI IV 2a  OpenBMI | NR | Paired t-test | √ | NR | **√** | √ | √ | NR | NR | NR | Medium |
|  | *Zhu et al., 2024* | NR | BCI IV 2a  BNCI2015001 | https://github.com/grilled-chickenburger/bci | NR | √ | NR | **√** | √ | √ | NR | NR | N/A | Medium |
|  | *Nagarajan et al.,2024* | NR | OpenBMI  Stroke Dataset | NR | Wilcoxon signed-rank test | × | NR | **√** | √ | √ | NR | NR | NR | Medium |
|  | *Yu et al., 2024* | MBCNN-TCN | BCI IV 2a | NR | NR | √ | NR | **√** | √ | √ | NR | NR | NR | Low |
|  | *Khabti, AlAhmadi & Soudani, 2024* | FCNNA | BCI IV 2a | NR | NR | × | N/A | **√** | √ | √ | NR | NR | NR | Medium |
|  | *Li et al., 2024a* | SSCL-CSD | BCI IV 2a  BCI IV 2b  HGD | NR | Wilcoxon signed-rank test | √ | N/A | **√** | √ | √ | NR | NR | N/A | Low |
|  | *Sun et al., 2024* | CLS | GIST  OpenBMI | NR | Wilcoxon signed-rank test | √ | N/A | **√** | √ | √ | NR | NR | N/A | Medium |
|  | *Wu, Li & Wu, 2024* | SWPC | BCI IV 2a  BCI IV 2b  BNCI2014002 | https://github.com/why135724/SWPC | Paired t-test | √ | N/A | **√** | √ | √ | NR | NR | N/A | Medium |
|  | *Olvera et al., 2024* | QGA | BCI IV 2b | NR | Paired t-test | NR | N/A | **×** | √ | √ | NR | NR | N/A | Medium |
| 2025 | *Zhi et al., 2025* | SCLDGN | BCI IV 2a  High Gamma  GIST  OpenBMI  PhysioNet | https://github.com/hongyizhi/SCLDGN. | Wilcoxon signed-rank test | √ | N/A | **√** | √ | √ | √ | √ | N/A | Low |
|  | *Xu et al., 2025* | CLUDA | BCI IV 2a  BCI IV 2b  SHU  OpenBMI | NR | Wilcoxon signed-rank test | √ | √ | **√** | √ | √ | NR | NR | × | Medium |
|  | *Li et al., 2025* | MIFM | BCI IV 2a  in-house dataset | NR | NR | √ | NR | **√** | √ | √ | NR | NR | × | Low |
|  | *Yang et al., 2025a* | JFACPS | BCI IV 2a  BCI IV 2b  OpenBMI  SHU | NR | Paired t-test | √ | √ | **√** | √ | √ | √ | NR | × | Medium |
| 2025 | *Wang et al., 2025b* | EEGMamba | PhysioNet | https://github.com/wjq-learning/EEGMamba | NR | √ | N/A | **√** | √ | √ | NR | NR | N/A | Medium |
|  | *Wang et al., 2025c* | DBConformer | BCI IV 2a  BCI IV 2b  Zhou2016  BCI III 4a  SMR-BCI  OpenBMI | https://github.com/wzwvv/ DBConformer | Paired t-test | √ | N/A | **√** | √ | √ | √ | NR | N/A | High |
|  | *Jin et al., 2025* | MSTFNet | BCI IV 2a  BCI IV 2b  in-house dataset | NR | Paired t-test | √ | N/A | **√** | √ | √ | NR | NR | N/A | Medium |

^1^ √: Yes; ×: No; NR: Not Reported; N/A: Not Applicable.
